# Challenges and Strategies of Chemical Analysis of Drugs of Abuse and Explosives by Mass Spectrometry

**DOI:** 10.3389/fchem.2020.598487

**Published:** 2021-01-18

**Authors:** Ahsan Habib, Lei Bi, Huanhuan Hong, Luhong Wen

**Affiliations:** ^1^The Research Institute of Advanced Technologies, Ningbo University, Ningbo, China; ^2^Department of Chemistry, University of Dhaka, Dhaka, Bangladesh; ^3^China Innovation Instrument Co., Ltd., Ningbo, China

**Keywords:** drugs of abuse, explosives, ambient ionization source, hollow cathode discharge ionization, headspace method, non-thermal desorption, tribological effect, mechanism of ionization and desorption

## Abstract

In analytical science, mass spectrometry (MS) is known as a “gold analytical tool” because of its unique character of providing the direct molecular structural information of the relevant analyte molecules. Therefore, MS technique has widely been used in all branches of chemistry along with in proteomics, metabolomics, genomics, lipidomics, environmental monitoring etc. Mass spectrometry-based methods are very much needed for fast and reliable detection and quantification of drugs of abuse and explosives in order to provide fingerprint information for criminal investigation as well as for public security and safety at public places, respectively. Most of the compounds exist as their neutral form in nature except proteins, peptides, nucleic acids that are in ionic forms intrinsically. In MS, ion source is the heart of the MS that is used for ionizing the electrically neutral molecules. Performance of MS in terms of sensitivity and selectivity depends mainly on the efficiency of the ionization source. Accordingly, much attention has been paid to develop efficient ion sources for a wide range of compounds. Unfortunately, none of the commercial ion sources can be used for ionization of different types of compounds. Moreover, in MS, analyte molecules must be released into the gaseous phase and then ionize by using a suitable ion source for detection/quantification. Under these circumstances, fabrication of new ambient ion source and ultrasonic cutter blade-based non-thermal and thermal desorption methods have been taken into account. In this paper, challenges and strategies of mass spectrometry analysis of the drugs of abuse and explosives through fabrication of ambient ionization sources and new desorption methods for non-volatile compounds have been described. We will focus the literature progress mostly in the last decade and present our views for the future study.

## Introduction

Mass spectrometry (MS) is one of the most powerful and widely used modern physical-chemical methods for analyzing all types of compounds and most of the elements in the periodic table with high selectivity and sensitivity. In analytical science, MS is known as a “gold standard” because of its unique character of providing the direct molecular structural information of the compounds of interest. Therefore, MS technique has been widely used in all branches of chemistry along in proteomics, metabolomics, genomics, lipidomics, environmental monitoring etc. and also in technology. MS-based techniques have already been proven as a versatile analytical tool for solving many analytical problems such as characterization of biomolecules such as proteins, peptides, nucleic acids and also analysis of polymers. MS has widely been used to elucidate the structure of the compounds of interest through fragmentation using tandem MS (MS^2^) as well. In fact, MS technique is the method of choice for industrial, clinical, biological, forensic, environmental monitoring, isotope etc. analyses. The development of the MS-imaging system is a milestone to the scientific world because of its precise medical diagnosis such as tumor, cancer etc. through characterization of complex biomolecules (Rohner et al., 2005; McDonnell and Heeren, 2007; Addie et al., 2015).

Furthermore, application of MS in space research that started in the 1950's by the US Naval Research Laboratory has already been proven its high-throughput as an indispensable analytical tool in science and technology (Robert, 2008; Arkin et al., 2010). Mass spectrometry has been used by the International Space Station for monitoring air quality, propellant leakage of the Space Shuttles etc. (NASA, 2007). Japan Aerospace Exploration Agency (JAXA) has already been taken a collaborative research with the Indian Space Research Organization (ISRO) to send the H3-launch vehicle and the rover called as lunar polar exploration mission that for searching the existence of water to the south pole region of the moon in 2024 (Hoshino et al., 2019, 2020; The Yomiuri Shimbun, 2019). It is noted that the outstanding analytical performance of MS-based techniques facilitated us to achieve adequate knowledge and understanding about the solar system as well as the universe.

Applications of the MS technique should be emphasized into two general types: (i) detection and characterization of compounds introduced into the MS where it can be considered as a powerful detector and (ii) investigation of physical and/or chemical transformations of the compounds being studied. In the former case, MS couples directly with an ionization source for detection and quantification of the relevant analytes (Fenn et al., 1989; Gale and Smith, 1993; McLuckey et al., 1996; Feng and Smith, 2000; Byrdwell, 2001; Cody et al., 2005; Shiea et al., 2005; Takáts et al., 2005; Hiraoka et al., 2006, 2007, 2015; Song and Cooks, 2006; Ganeev et al., 2007; Na et al., 2007a,b; Cotte-Rodríguez et al., 2008; Harper et al., 2008; Zhang et al., 2009; Chen et al., 2010, 2017; Nilles et al., 2010; Garcia-Reyes et al., 2011; Takada et al., 2011; Habib et al., 2013, 2014, 2015, 2020; Sekar et al., 2013; Su et al., 2013; Groeneveld et al., 2015; Castiglioni et al., 2016; Damon et al., 2016; Kumano et al., 2018; Tavares et al., 2018; Usmanov et al., 2018; Borges et al., 2019; Feider et al., 2019; Ng et al., 2019) and/or couple with a separation technique (chromatographic) such as gas chromatography (GC), liquid chromatography (LC), high performance liquid chromatography (HPLC), electrophoresis, paper chromatography etc. and then coupled with an ionization source for ionization (Horning et al., 1974; Carroll et al., 1975; Alexandrov et al., 1984; Whitehouse et al., 1985; Olivares et al., 1987; Smith et al., 1988a,b; Lee et al., 1989; Covey and Devanand, 2002; Sigman et al., 2006; Pizzolato et al., 2007; Khan et al., 2015; Gilbert-López et al., 2019). Mass spectrometer comprises an ion source, mass analyzer and a detector. In MS, ion source is considered as the heart of it because the sensitivity mostly depends on the ionization efficiency of the ion source. Since the discovery of MS in the 1950's, electron ionization (EI) (Dempster, 1918, 1921), chemical ionization (CI) (Fales et al., 1972; Harrison, 1992; Field, 2002), fast atom bombardment (FAB) ionization (Barber et al., 1981a,b, 1982), Panning ionization (Penning, 1927; Arango et al., 2006; Hiraoka et al., 2006) were widely used for ionization of the compounds of interest under vacuum condition. In the 1970s, Carroll and co-workers at the Baylor College of Medicine invented the atmospheric pressure chemical ionization (APCI) (Horning et al., 1973) that was applied to gas chromatography-mass spectrometry (GC-MS) (Carroll et al., 1975) and liquid chromatography-mass spectrometry (LC-MS) (Horning et al., 1974). In 1975, a corona discharge electrode developed that has widely been used as an APCI ion source for commercially available MS system (Byrdwell, 2001).

In the history of MS, development and application of electrospray ionization (ESI) opened a new research arena for the analysis of macromolecules and biological materials (Whitehouse et al., 1985; Meng et al., 1988; Smith et al., 1988a, 1990; Fenn et al., 1989, 1990, 1997; Lee et al., 1989; Loo et al., 1990; Cole, 1997; Kebarle, 2000). Electrospray ionization (ESI) is a technique that uses high voltage to generate ions from an aerosol of charged liquid droplets. Since its first discover by Rayleigh (1882) for theoretical estimation of maximum amount of charge of a liquid droplet could carry before throwing out fine jets of liquid that is known as a Rayleigh limit (Rayleigh, 1882) and has then extended by Zeleny (1914) to study the behavior of fluid droplets at the end of glass capillaries for knowing different electrospray modes (Zeleny, 1914). After that, Wilson and Taylor (Wilson and Taylor, 1925) and (Nolan and O'Keeffe, 1932) investigated electrospray extensively in the 1920s. The electrospray cone was described by Sir Geoffrey Ingram Taylor (Taylor, 1964), accordingly, the cone later coined as Taylor cone (Wilm and Mann, 1994). Macky (1931) also studied the behavior of electrospray (Macky, 1931). Dole et al. (1968) used the electrospray ionization with MS for the first time in 1968 (Dole et al., 1968; Pramanik et al., 2002) and finally Yamashita and Fenn (Yamashita and Fenn, 1984; Whitehouse et al., 1985; Meng et al., 1988; Fenn et al., 1989, 1990, 1997) as well as Alexandrov et al. (1984) and co-workers developed the electrospray ionization mass spectrometry in 1984. John B. Fenn was awarded the Nobel Prize for the development of ESI-MS in Chemistry in 2002 (Press Release, 2002).

In fact, commercial ESI utilizes high flow rates that keep within a range from 10.0 μL/min to 1.0 mL/min (Meng et al., 1988; Smith et al., 1988a,b, 1990; Fenn et al., 1989, 1990; Lee et al., 1989; Loo et al., 1990; Kebarle, 2000; Sekar et al., 2013; Abdelhamid and Wu, 2015, 2019a; Khan et al., 2015). The high flow rate is responsible for the formation of liquid droplets with large size that causes slow evaporation, thereby resulting in obtaining poor sensitivity of the relevant analyte compounds. The high flow rate system also consumes large sample volume that causes less stability of the spray. However, when the flow rate is reduced to nanoliters per minute (nL/min) in case of nano-ESI, droplet formation occurs more readily, requiring only the applied voltage to the metal-coated nano-capillary (Olivares et al., 1987; Smith et al., 1988a; Gale and Smith, 1993; Wilm and Mann, 1996; Juraschek et al., 1999; Feng and Smith, 2000; Karas et al., 2000; Covey and Devanand, 2002; Ushijima et al., 2019). Accordingly, the spray achieves stability with relatively smaller size of the droplets that results in better sensitivity. Though nano-ESI exhibits better sensitivity than that by ESI, however, nano-ESI has been limited in its applications for biological fluids because of clogging problems by the presence of high salts and/or other biological. To overcome the clogging problem, Hiraoka and co-workers developed a needle-based ESI coined as a probe-electrospray (PESI) ion source for MS that can operate under ambient conditions (Hiraoka et al., 2007, 2015; Usmanov et al., 2018). In ambient mass spectrometry, ionization of neutral compounds of interest occurs under ambient conditions and then collected and analyzed by MS (Takáts et al., 2004; Cody et al., 2005; Cooks et al., 2006; Feider et al., 2019). The ambient ion sources allow the high-throughput analysis of sample of interest in their native state, often with minimal or no sample pre-treatment. The developed ambient ion sources such as desorption electrospray ionization (DESI) (Takáts et al., 2004; Justes et al., 2007), direct analysis in real time (DART) (Cody et al., 2005), matrix-assisted laser desorption/ionization (MALDI) (Karas et al., 1985; Abdelhamid and Wu, 2012, 2013, 2016, 2019a,b; Abdelhamid et al., 2017; Chen et al., 2017; Abdelhamid, 2018, 2019a,b), electrospray-assisted laser desorption/ionization (ELDI) (Shiea et al., 2005), desorption atmospheric pressure chemical ionization (DAPCI) (Takáts et al., 2005) have already been coupled to commercial MS. Beside these, ac-atmospheric pressure chemical ionization (ac-APCI) (Habib et al., 2013), He-flow dielectric barrier discharge ionization (DBDI) (Na et al., 2007a,b; Zhang et al., 2009; Habib et al., 2014, 2020; Gilbert-López et al., 2019), PESI (Hiraoka et al., 2007) have been fabricated as ambient ion sources and applied to analyze a wide range of compounds. Since the pioneering work of the DBD ion source by Zhang and co-workers (Na et al., 2007a,b; Zhang et al., 2009), it has widely been used in mass/ion-mobility spectrometry (Usmanov et al., 2013; Habib et al., 2014, 2020; Gilbert-López et al., 2019). According to the design of the homemade He-DBDI, H_3_O^+^ and ${\text{NO}}_{2}^{-}$ and/or ${\text{NO}}_{3}^{-}$ ions are formed as reagent ions in positive and negative ion modes, respectively and the gaseous neutral molecules of interest take part in ion-molecule reactions outside of the plasma. In the positive ion mode, the gaseous neutral analyte molecules ionized mainly by the H_3_O^+^, however, in the negative ion mode, the reagent ions ${\text{NO}}_{2}^{-}$ and/or ${\text{NO}}_{3}^{-}$ combine with the analyte molecules lead to cluster and/or adduct ions (Usmanov et al., 2013; Habib et al., 2014, 2020). In our fabricated DBDI, water forms its cluster ion, H_3_O^+^(H_2_O)_n_, outside of the DBD ceramic tube, accordingly, it is regarded as an ambient ion source just mimic of APCI.

In analytical mass spectrometry, it is highly appealing that the investigated analytes should undergo minimal fragmentation during desorption/ionization in order to achieve better limits of detection (LODs). Among the developed ion sources, MALDI, DESI, ESI, and/or PESI have been proved as soft ionization methods for a wide range of compounds ranging from large molecules such as proteins, peptides, polymers, nucleic acids etc. to small molecules like amino acids, drugs of abuses, explosives and many of commonly naturally found compounds. As mentioned, an ionization method plays a vital role in quantifying the relevant analytes at ultra-trace level where efficient desorption of the investigated compounds is mandatory. In this regard, MALDI, DESI, or ESI function both as an efficient ion source and desorption method simultaneously. Since the pioneering work of MALDI by Franz Hillenkamp, Michael Karas, and their colleagues in 1985, much attention has been paid to develop matrices for efficient desorption and ionization of particularly biomolecules such as peptides, proteins, nucleic acids, lipids, oligosaccharides, amino acids etc. (Karas et al., 1985, 1987; Tanaka et al., 1988; Karas and Bahr, 1990; Fitzgerald et al., 1993; Tang et al., 1994; Abdelhamid and Wu, 2012, 2013, 2016, 2019a,b; Abdelhamid et al., 2017; Chen et al., 2017; Abdelhamid, 2018, 2019a,b). Organic compounds such as 2,5-dihydroxy benzoic acid, 3,5-dimethoxy-4-hydroxycinnamic acid, 4-hydroxy-3-methoxycinnamic acid, α-cyano-4-hydroxycinnamic acid, picolinic acid, and/or 3-hydroxy picolinic acid etc. have widely been used as MALDI matrices for desorption/ionization of peptides, proteins, nucleotides, oligonucleotides, oligosaccharides, lipids etc. (Karas et al., 1985, 1987; Tanaka et al., 1988; Beavis et al., 1989, 1992; Karas and Bahr, 1990; Strupat et al., 1991; Fitzgerald et al., 1993; Wu et al., 1993), however, inorganic materials, particularly nanoparticles (NPs) have advanced applications in mass spectrometry as MALDI matrices (Abdelhamid and Wu, 2012, 2013, 2016, 2019a,b; Abdelhamid et al., 2017; Chen et al., 2017; Abdelhamid, 2018, 2019a,b). Besides the development of hybrid desorption/ionization system, attentions have also been taken to fabricate desorption method for non-volatile compounds from their solid state (Saha et al., 2013; Usmanov et al., 2013, 2016; Habib et al., 2014; Bi et al., 2021). Of them, Leidenfrost-assisted thermal desorption (LPTD) and ultrasonic-cutter blade-based desorption methods exhibited efficient desorption methods for gasification of mostly non-volatile compounds without and/or with minimal fragmentation (Saha et al., 2013; Usmanov et al., 2013, 2016; Habib et al., 2014; Bi et al., 2021). Coupling of these noble desorption methods with homemade helium-DBDI-MS system exhibited as potential analytical techniques in detection and quantification of highly non-volatile drugs of abuse, explosives, carbohydrates, rhodamine B, ionic liquids, amino acids, spinosad etc. at ultra-trace levels to some extent (Saha et al., 2013; Habib et al., 2014, 2020; Bi et al., 2021).

The direct structural information of compounds of interest using mass spectrometry has given golden opportunity to the scientists and researchers of its versatile applications in various fields in science and technology. Accordingly, MS technique in forensic laboratories as well as for explosive detection has been increased significantly in the last few decades from criminal investigation and public security and safety point of views. The sensitivity, rapid analysis, selectivity and simple sample pretreatment requirements have led to MS-based methods being adopted as the choice to analyze illicit drugs (Pizzolato et al., 2007; Habib et al., 2014, 2020; Groeneveld et al., 2015; Damon et al., 2016; Brettell and Lum, 2018; Tavares et al., 2018; Borges et al., 2019; Ng et al., 2019; Filho et al., 2020) and explosives (Chen et al., 2009; Takada et al., 2011, 2012a,b, 2016; Habib et al., 2013, 2014; Hashimoto et al., 2014; Kumano et al., 2018). Besides these applications, MS-based technique has also been used in analysis of complex biological samples through coupling with micro-chips, microfluidic surface extractor (MSE) and/or single drop microextraction device (He et al., 2014; Chen et al., 2017; Liu et al., 2020; Zhang et al., 2020).

**Scheme 1:**
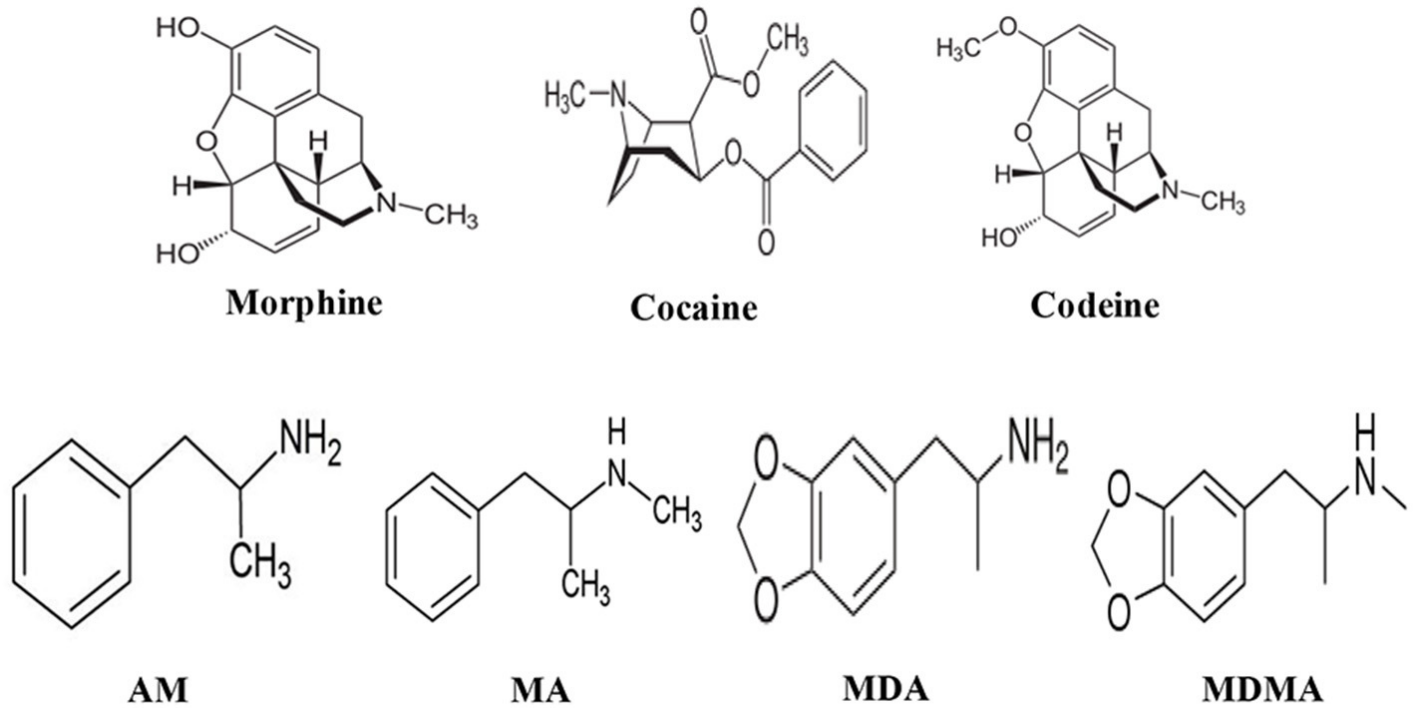
Molecular structures of the illicit drug compounds.

**Scheme 2:**
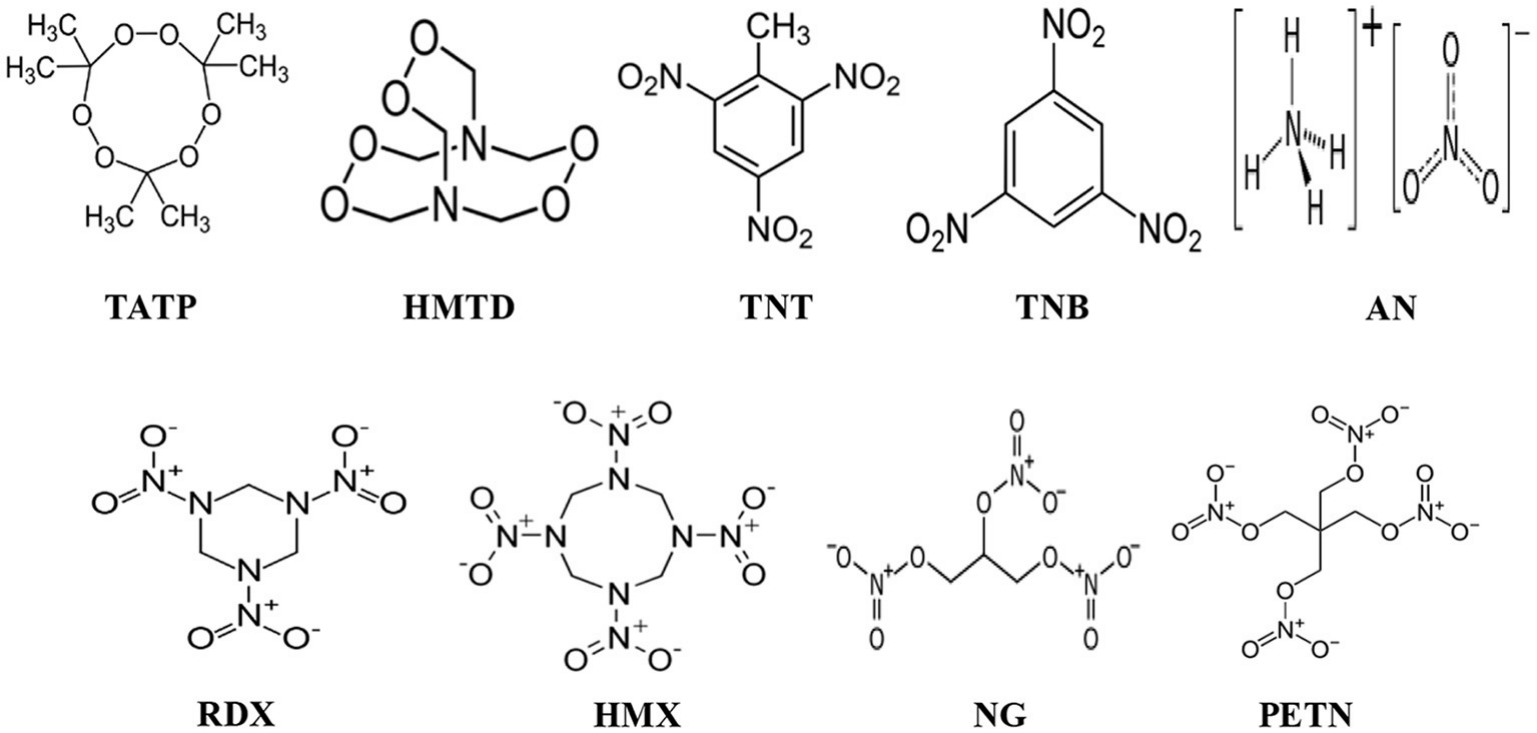
Molecular structures of the explosive compounds.

In this review article, challenges and strategies of mass spectrometry analysis of the illicit drugs and explosives through fabrication of new ionization sources and new desorption methods for non-volatile compounds have been described. As illicit drugs, morphine, cocaine, codeine, amphetamine (AM), methamphetamine (MA), 3,4-methylenedioxyamphetamine (MDA), and 3,4-methylenedioxymethamphetamine (MDMA) (Scheme 1) and as explosives, triacetone triperoxide (TATP), hexamethylene triperoxide diamine (HMTD), trinitrotoluene (TNT), 1,3,5-trinitrobenzene (TNB), ammonium nitrate (AN), hexahydro-1,3,5-trinitro-1,3,5-triazine (RDX), 1,3,5,7-tetranitro-1,3,5,7-tetrazoctane (HMX), nitroglycerine (NG), and pentaerythritol tetranitrate (PETN) (Scheme 2) were taken as model compounds. In view of this, firstly, the following ionization sources: dielectric barrier discharge ionization (DBDI), hollow cathode discharge ionization (HCDI), ac/dc-atmospheric pressure chemical ionization (APCI) secondly, thermal/non-thermal desorption methods for non-volatile compounds such as solid/solid friction and flash heating/rapid cooling have been described. The developed DBDI and ac/dc-APCI ion sources function well under ambient conditions while the HCDI works well at much below atmospheric (ranging from 1 to 28 Torr) pressure from sensitivity and selectivity point of views. Mechanisms of ion formation and desorption of non-volatile drug and explosive compounds by using the developed ion sources and solid/solid-based desorption method are also discussed.

## Tribodesorption-Dielectric Barrier Discharge (DBD) Ionization-MS Syetm

### Concept of Desorption of Non-volatile Compounds by Tribological Effect

**Figure 1:**
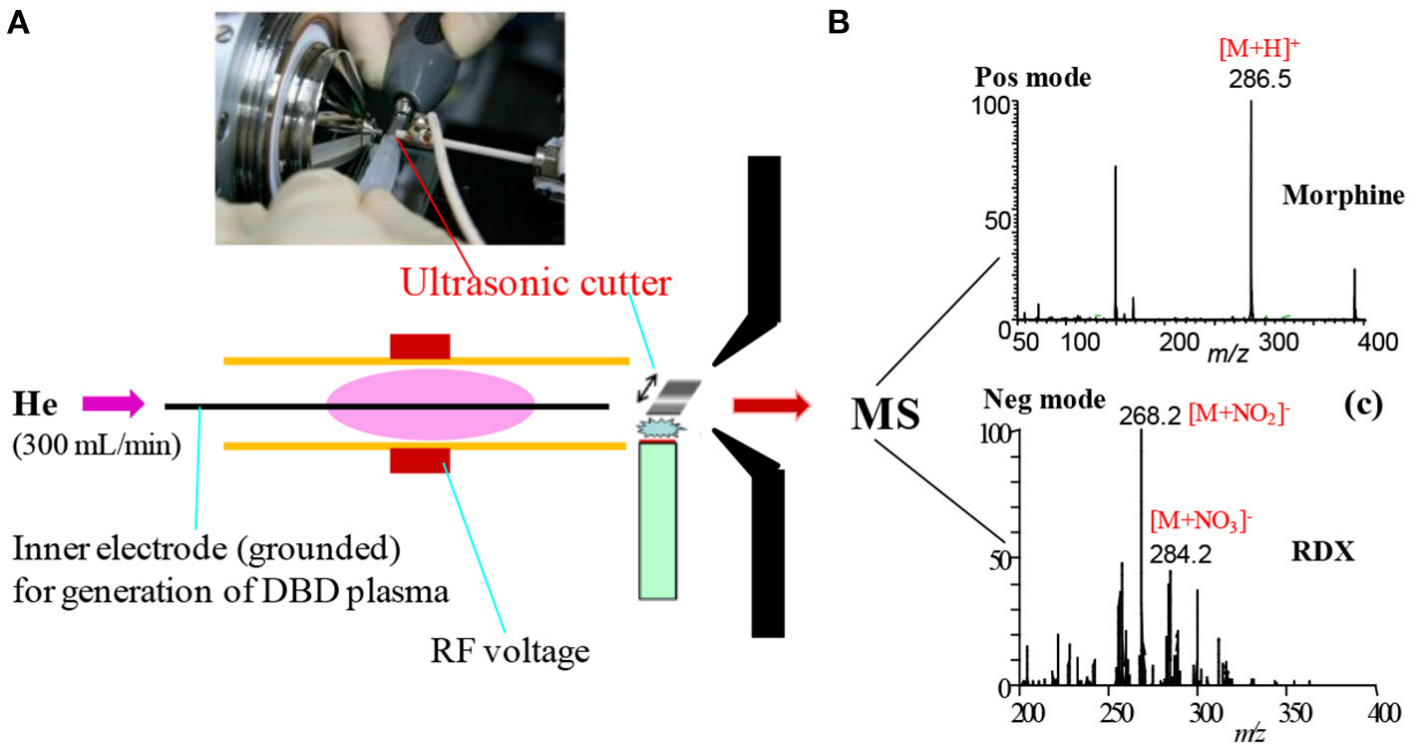
A diagram of homemade He-DBD ion source coupled with MS **(A)**, mass spectrum of morphine in positive ion mode **(B)** and mass spectrum of RDX in negative ion mode **(C)**. An ultrasonic cutter-assisted desorption method was used for desorption of the non-volatile illicit drugs and explosives. The inset is the photograph of ultrasonic cutter-PFA solid/solid experimental system. Reproduced under permission from ACS (Habib et al., 2014).

Figure 1 shows a homemade helium dielectric barrier discharge (DBD) ion source fabricated using a ceramic tube as a dielectric barrier tube where a stainless steel wire was inserted into the ceramic tube and grounded that acted as an inner electrode (Usmanov et al., 2013; Habib et al., 2014, 2020; Bi et al., 2021). The DBD plasma was generated inside the ceramic tube by flowing He gas (99.9999%) with a flow rate ranging from 0.25 to 0.30 L/min through applying 2.4 kV_PTP_ (peak-to-peak) ac high voltage to the outer electrode (copper strip). A function generator was used to generate 15 kHz sinusoidal signal and then amplified by a power generator in order to generate the ac high voltage. The inner grounded wire electrode was protruded from the exit of the ceramic tube, thereby resulting in confinement of the DBD plasma inside the ceramic tube. As a result, the desorbed gaseous analyte molecules were not exposed directly by the plasma but ionized by reacting with mainly H_3_O^+^ ion and/or with ${\text{NO}}_{2}^{-}$/${\text{NO}}_{3}^{-}$ in the positive and negative modes of ionization, respectively. Under the ambient condition, water forms its cluster ion, H_3_O^+^(H_2_O)_n_, outside of the DBD ceramic tube. The present DBD ion source is thus regarded as an atmospheric pressure chemical ionization (APCI) (Usmanov et al., 2013; Habib et al., 2014, 2020; Bi et al., 2021).

**Figure 2:**
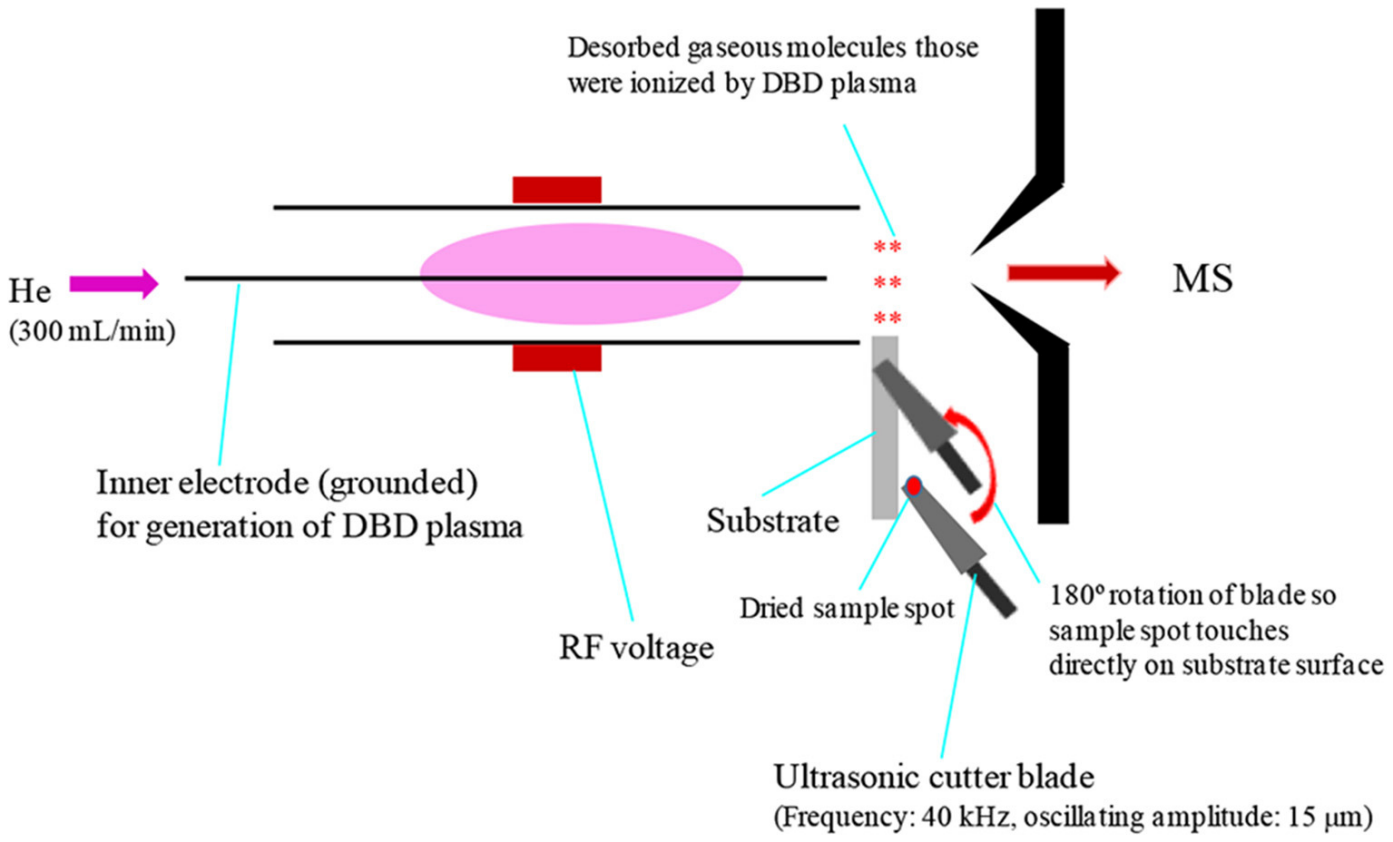
A schematic of experimental set-up for tribodesorption-dielectric barrier discharge ionization-mass spectrometry (TB-DBDI-MS) system. The sample deposited on the ultrasonic blade and gently touches on PFA substrate's surface by hand holding (frequency: 40 kHz, oscillating amplitude: 15 μm) for desorption. A homemade He-DBD ion source was used to ionize the analyte molecules and then characterized by using an ion trap mass spectrometer.

Prior to discussing the mass spectral results, it is better to focus on the mechanism of desorption of the non-volatile analyte compounds by the ultrasonic cutter-based desorption method. In this desorption method, exactly 2 μL of the sample solution was deposited on a flat perfluoroalkoxy (PFA) substrate and diameter of the sample spot was found to be 2 mm. After dried in air, the deposited sample on the PFA substrate was desorbed through very gentle touching with an ultrasonic cutter by applying an oscillation frequency of 40 kHz to the cutter blade (20 W, Honda Electronics, Toyohashi, Japan). The gaseous molecules were then ionized using a homemade He-DBDI and detected by an ion trap mass spectrometer. The solid/solid friction of the ultrasonic cutter with the PFA substrate has been coined as a tribological event (Habib et al., 2014; Bi et al., 2021). In another study, we found enhanced sensitivity of the relevant compounds when the compounds were deposited on the ultrasonic blade instead of the substrate and then gently touch with the substrate's surface for rubbing (Bi et al., 2021). Such an experimental setup is shown in Figure 2. This is because the oscillation/vibrational energy from the ultrasonic cutter blade directly transfer to the deposited solid molecules as an additional kinetic energy that causes the intermolecular force of attraction to weaken before touching the substrate. During the touching/rubbing process, the deposited molecules on the blade also gain mechanical/frictional energy in terms of tribological event as an extra energy in order to amplify the desorption process before penetrating and/or remaining into/on the substrate. This synergistic effect, caused by gaining the vibrational/oscillation and frictional/mechanical energy, enhances the desorption process of the non-volatile solid molecules into the gaseous phase that provides better sensitivity, realizing ultra-trace level detection.

The term tribology means the interface of moving matters. Friction of moving bodies also plays a vital role in the interface. In MS, analytes in gaseous phase is mandatory consequently, thermal energy has long been used for desorption of solid molecules into the gaseous phase, however, thermal energy may cause fragmentation of the analyte compounds that is not expected in analytical mass spectrometry. This is because fragmentation may not provide better limits of detection in most of the MS experiments while that is highly desirable in analytical MS. Mechanical energy in terms of tribological event is commonly found in nature as well as in many scientific and technological areas. However, thermal energy and mechanical energy are inherently different. The former is a scalar quantity with random energy while the latter is a vector quantity where all the atoms and molecules of the moving matters have the same direction. Therefore, it may conclude that mechanical energy is regarded as the high-quality energy with coherency. The macroscopic mechanical energy is transferred to non-thermal energy by the nano- and/or microscopic contact with the moving bodies, thus it seems very difficult to recognize molecular-level information by the conventional analytical techniques (Nevshupa, 2009). Mass spectrometry is a highly appealing analytical tool because of its direct structural information of the generated molecules and/or ions during moving bodies, ultimately interface mechanism will be achieved.

**Figure 3:**
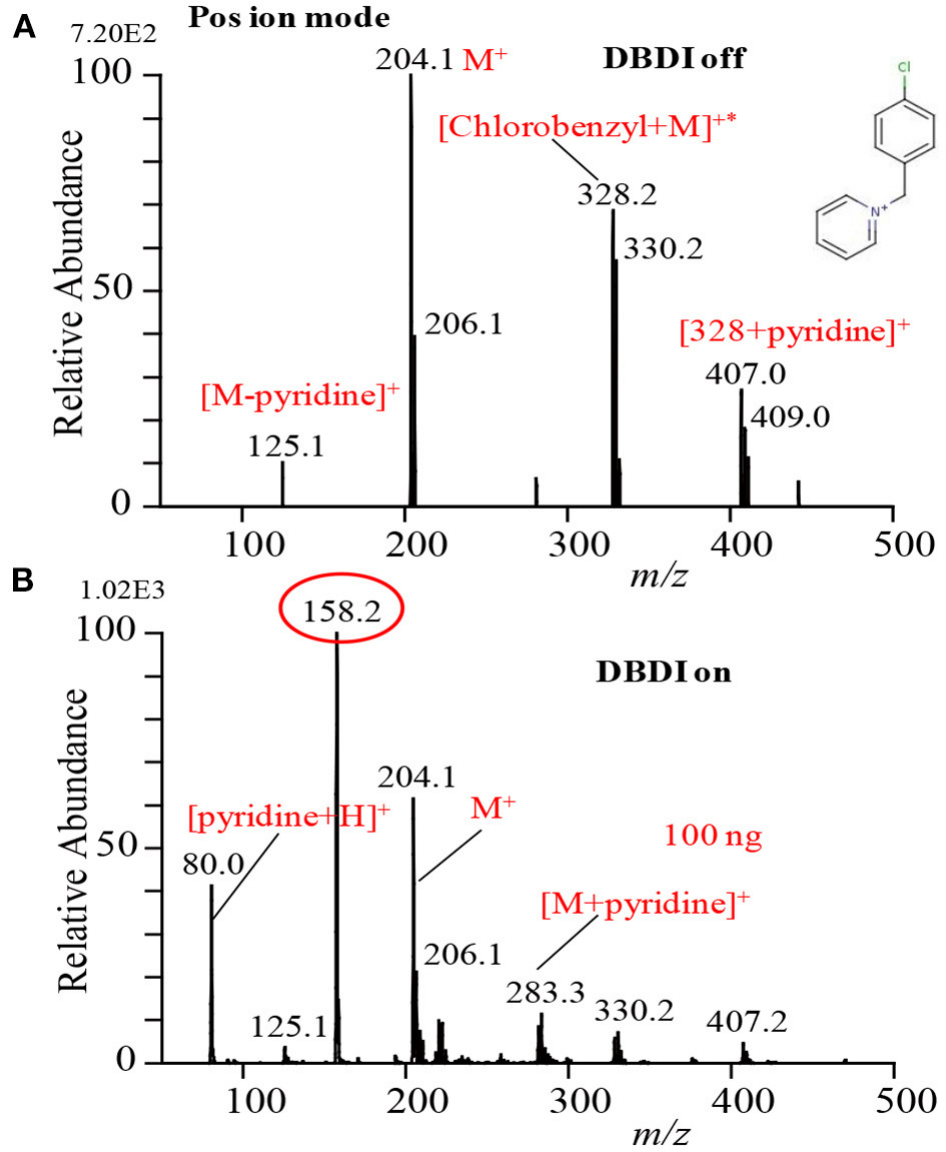
Mass spectra for p-chloro-benzyl pyridinium chloride with DBD turned **(A)** off and **(B)** on. Reproduced under permission from ACS (Habib et al., 2014).

In order to investigate whether the ultrasonic cutter-based desorption method is non-thermal or thermal, p-chloro-benzyl pyridinium chloride was taken as a model thermometer compound to obtain direct information about desorption (Habib et al., 2014). The p-chloro-benzyl pyridinium chloride is a highly non-volatile ionic compound. Figure 3 shows the mass spectrum of the p-chloro-benzyl pyridinium chloride in the positive mode of ionization. The compound gave molecular ion, M^+^, as the major ion that appeared at *m/z* 204.1 along with a much weaker signal from the fragment ion [M-pyridine]^+^ (i.e., p-chlorobenzyl cation, ClC_6_H_4_${\text{CH}}_{2}^{+}$, *m/z* 125.1) as the DBDI was turned off condition. Therefore, it is concluded that the fabricated ultrasonic cutter-based desorption method can efficiently gasify highly non-volatile compounds as their monomer with a very minimal fragmentation. It has also been reported that the temperature of effused gases just outside of the dielectric ceramic tube was to be ~49°C that was slightly elevated from room temperature (Habib et al., 2014). The radiofrequency heating of the dielectric tube causes such elevation of the gas temperature. On the other hand, the direct analysis in real time (DARD) fabricated by Cody and co-workers used quite high gas temperature in order to gasify the solid analyte compounds for ionization (Cody et al., 2005).

In conclusion, the soft mechanical frictional energy generated by the ultrasonic cutter in combination with a substrate can be used as an efficient desorption process for gasification of non-volatile compounds. The developed method, especially deposition of the relevant compound on the cutter blade instead of the substrate (Bi et al., 2021), is reasonably sensitive and may be applied for quick analysis of contaminants, such as narcotics, explosives, and/or any other non-volatile compounds, deposited on substrates' surfaces.

### Analysis of Drugs of Abuse

**Figure 4:**
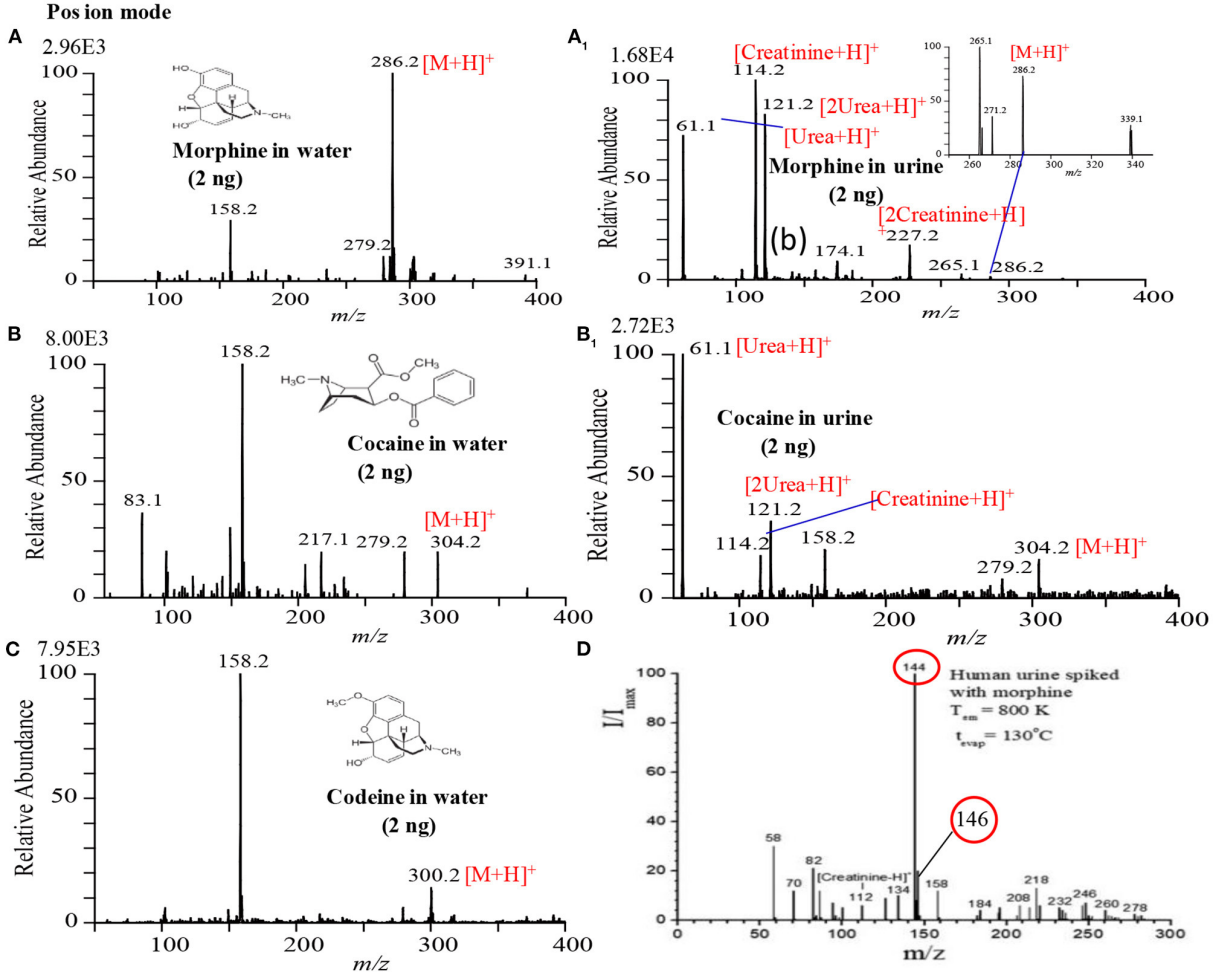
Mass spectra for **(A)** 2 ng morphine, **(A1)** 2 ng morphine in urine, **(B)** 2 ng cocaine, **(B1)** 2 ng cocaine in urine, **(C)** 2 ng codeine and **(D)** morphine in urine. Each of the illicit drugs was deposited on a perfluoroalkoxy (PFA) substrate and then rubbed by an ultrasonic cutter blade for desorption. The gaseous molecules were then ionized using a homemade helium DBDI source and detected by MS. Reproduced under permission from ACS (Habib et al., 2014) and EJMS (Usmanov et al., 2020).

Figure 4 shows the mass spectra of morphine, cocaine and codeine. Exactly 2 μL sample solution containing 2 ng of each illicit drug compound was deposited on the PFA substrate and then dried in air. The dried spot was gently touched using the ultrasonic cutter for gasification. A homemade He-DBDI coupled with a MS was used for ionization/detection of the illicit drug compounds. As seen from Figure 4, morphine, cocaine, and codeine were detected in their protonated forms, [M+H]^+^, at *m/z* 286, 304, and 300, respectively without any fragmentation. However, Usmanov et al. (2020) reported that morphine underwent severe fragmentation by using surface-ionization mass spectrometry and the major fragment ions appeared at *m/z* 144, 146 when detected from human urine spiked (Figure 4D) (Usmanov et al., 2020).

Figures 4A_1_,B_1_ show the raw urine sample spiked with 2 ng of morphine and cocaine, respectively. Both the morphine and codeine display their protonated ions at *m/z* 286 and 304, respectively without any fragmentation along with the protonated urea, creatinine, and their protonated dimmers. The developed desorption method exhibited its potentiality for gasification of the non-volatile illicit drug compounds from complex body fluids as well. As mentioned in the previous section, the homemade DBD ion source acts as an ambient atmospheric pressure chemical ionization (APCI). In the positive ion mode, hydronium ion, H_3_O^+^, comes from the DBD ion source as a reagent ion and takes part in ion-molecule reactions with the gaseous analyte molecules under the ambient condition, thereby resulting in the formation of protonated molecular ion, [M+H]^+^ of analytes of interest. The limits of detection (LODs) for the morphine, cocaine and codeine from water/methanol and spiked in urine are tabulated in Table 1. The values of LODs for morphine, cocaine and codeine in water/methanol were smaller than that spiked in urine and the values were 0.30, 0.50, and 0.60 ng in water/methanol and 0.50, 0.80, and 0.90 ng from spiked in urine, respectively (Table 1). The elevated values of LOD for the illicit drugs spiked in urine are due to ion suppression effects by the constituents of urine such as urea, creatinine etc. As seen from Figures 4A_1_,B_1_, protonated creatinine and/or urea, [M+H]^+^, exhibit as base peaks because of higher proton affinity of these urine constituents than that of the illicit drugs of interest. The low molar mass of the urine's constituents also causes efficient desorption of creatinine, urea etc. from the sample spot during rubbing by the ultrasonic cutter with the substrate. In our another study, however, we found enhanced sensitivity of the amphetaminic drug compounds such as amphetamine (AM), methamphetamine (MA), 3,4-Methylene dioxy amphetamine (MDA) and 3,4-Methyl enedioxy methamphetamine (MDMA) from spiked urine by an alkaline treated headspace method (Table 1) (Habib et al., 2020). The amphetaminic drug compounds contain either amine or imine groups, thus they exhibit volatile and/or semi-volatile in nature at room temperature. Accordingly, these nitrogenous compounds make their pills through treating with hydrochloric acid in order to provide stability. So, alkali treatment causes evaporation of the free base amphetaminic compounds into the gaseous phase from solution. Ammoniated carbonated alkali solutions such as sodium carbonate, potassium carbonate etc. provide better limits of detection even from spiked urine (Table 1) (Habib et al., 2020). Addition of alkali solution into the HCl-amphetaminic drug evolves gaseous carbon dioxide that acts as a carrier gas to bring the free base amphetaminic molecules from the liquid to the gaseous phase. Presence of trace level ammonia also enhances the sensitivity of the amphetaminic drug compounds using the headspace-DBDI-MS system (Habib et al., 2020).

**Table 1:** Limits of detection (LODs) for drugs of abuse and explosives measured by tribodesorption-helium-DBDI-MS and other MS techniques.

**Target Compounds**	**Characteristic ion (*m/z*)**	**LOD (pg) Mean ± SD**	**RSD (%)**
Morphine (285.34)	**[M+H]**^+^**(286)**	(40.38 ± 1.55)^a^^*^, (59.63 ± 5.39)^a^^**^, (300)^b^, (25 × 10^3^)^c^, (5 × 10^3^)^d^ (3 × 10^3^)^e^	(3.83)^a^^*^, (9.04)^a^^**^
Morphine (285.34) spiked in human urine	**[M+H]**^+^**(286)**	(500)^b^^*^ (1 × 10^3^)^b^^**^	-
Cocaine (303.35)	**[M+H]**^+^**(304)**	(20.00 ± 0.91)^a^^*^, (500)^b^, (7 × 10^3^)^c^, (5 × 10^3^)^d^, (15)^e^	(4.56)^a^^*^
Cocaine (303.35) spiked in human urine	**[M+H]**^+^**(304)**	(39.88 ± 1.65)^a^^*^ (800)^b^^*^ (500)^d^	(4.14)^a^^*^
Codeine (299.36)	**[M+H]**^+^**(300)**	(50.25 ± 1.85)^a^^*^, (600)^b^, (3 × 10^3^)^c^, (500)^d^, (150)^e^	(3.68)^a^^*^
Codeine (299.36) spiked in human urine	**M+H]**^+^**(300)**	(900)^b^^*^	-
AM (135.21)	**[M+H]**^+^**(136)**	(0.10)^h^^†^ (0.60)^h^^††^	(7.89)^h^^†^
AM (135.21) spiked in human urine	**[M+H]**^+^**(136)**	(0.05)^h^^†^	(6.23)^h^^†^
MA (149.24)	**[M+H]**^+^**(150)**	(0.10)^h^^†^ (0.60)^h^^††^	(6.67)^h^^†^
MA (149.24) spiked in human urine	**[M+H]**^+^**(150)**	(1 × 10^3^)^d^, (0.04)^h^^†^	(5.73)^h^^†^
MDA (179.22)	**[M+H]**^+^**(180)**	(0.80)^h^^†^ (3.00)^h^^††^	(14.05)^h^^†^
MDA (179.22) spiked in human urine	**[M+H]**^+^**(180)**	(0.40)^h^^†^	(8.56)^h^^†^
MDMA (193.25)	**[M+H]**^+^**(194)**	(0.60)^h^^†^ (1.00)^h^^††^	(11.72)^h^^†^
MDMA (193.25) spiked in human urine	**[M+H]**^+^**(194)**	(0.20)^h^^†^	(7.92)^h^^†^
HMTD (208.17)	**[M+H]**^+^**(209)**	(19.38 ± 0.85)^a^^*^, (30.25 ± 2.72)^a^^**^, (200)^b^, (3 × 10^3^)^d^	(4.41)^a^^*^, (9.00)^a^^**^
RDX (222.12)	**[M+NO**_**2**_**]**^**−**^**(268)** [M+NO_3_]^−^ (284)	(29.63 ± 1.31)^a^^*^, (50.75 ± 4.29)^a^^**^, (200)^b^, (5 × 10^3^)^c^, (50)^d^, (2 × 10^3^)^f^, (30)^g^	(4.44)^a^^*^, (8.46)^a^^**^
HMX (296.16)	**[M+NO**_**2**_**]**^**−**^**(342)** [M+NO_3_]^−^ (358)	(100.13 ± 4.66)^a^^*^, (900)^b^	(4.66)^a^^*^
AN (80.05)	**[HNO**_3_**+NO**_**3**_**]**^**−**^**(125)**	(200.25 ± 9.04)^a^^*^, (2 × 10^3^)^b^, (10 × 10^3^)^c^	(4.52)^a^^*^

*Reproduced under permission from ACS (Habib et al., 2014) and Elsevier (Bi et al., 2021)*.

a Bi et al. (2021) (Limits of detection in the present study (LODs: S/N = 3:1) and relative standard deviation (RSD %) using PFA* and wooden** substrate).

b *Habib et al. (2014)*.

c *Usmanov et al. (2013)*.

d *Usmanov and Hiraoka (2016)*.

e *Jackson et al. (2010)*.

f *Justes et al. (2007)*.

g *Garcia-Reyes et al. (2011)*.

h *Habib et al. (2020) (Limits of detection in the present study (LODs: S/N = 3:1) and relative standard deviation (RSD %) using (K2CO3+NH3)^†^ and (K2CO3)^††^ solutions)*.

*The limits of detection (LODs) of the compounds of interest have been estimated on the basis of the bold underlined peaks*.

**Figure 5:**
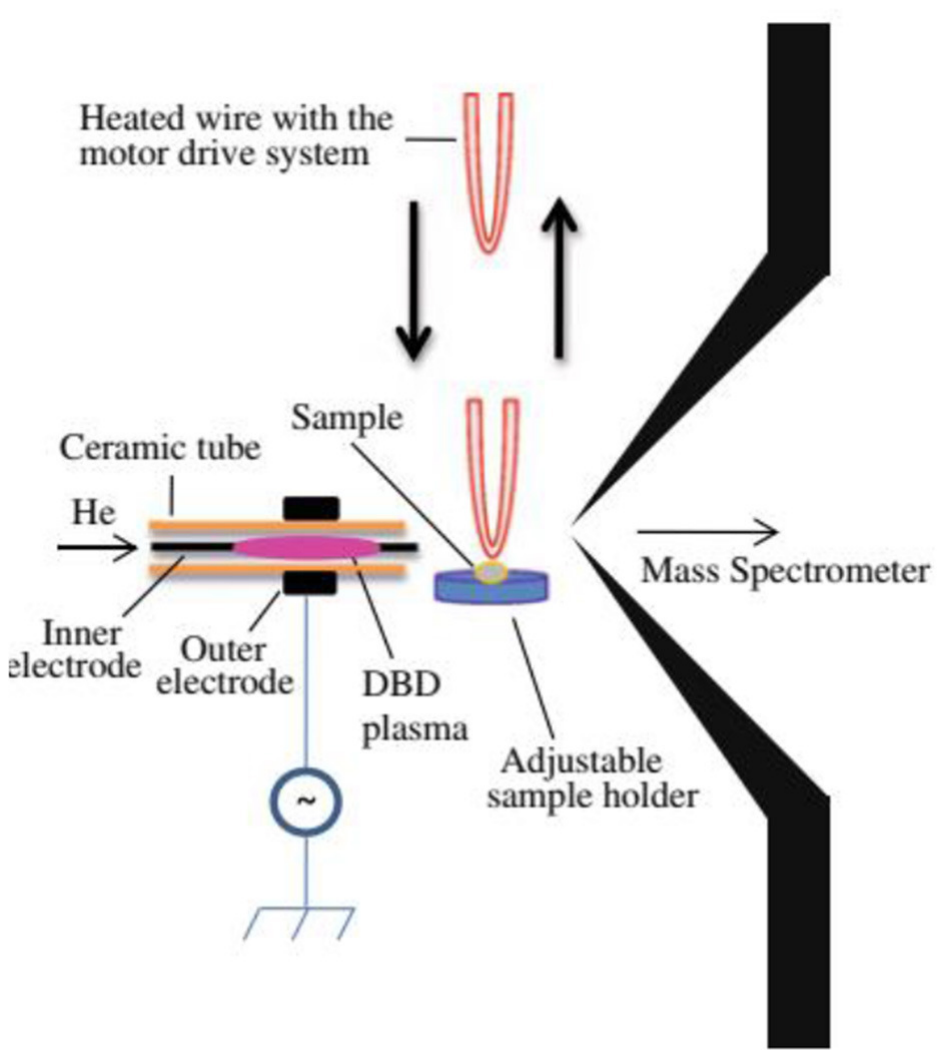
Schematic of the experimental setup for the heated filament-based desorption-DBDI-MS system.

**Figure 6:**
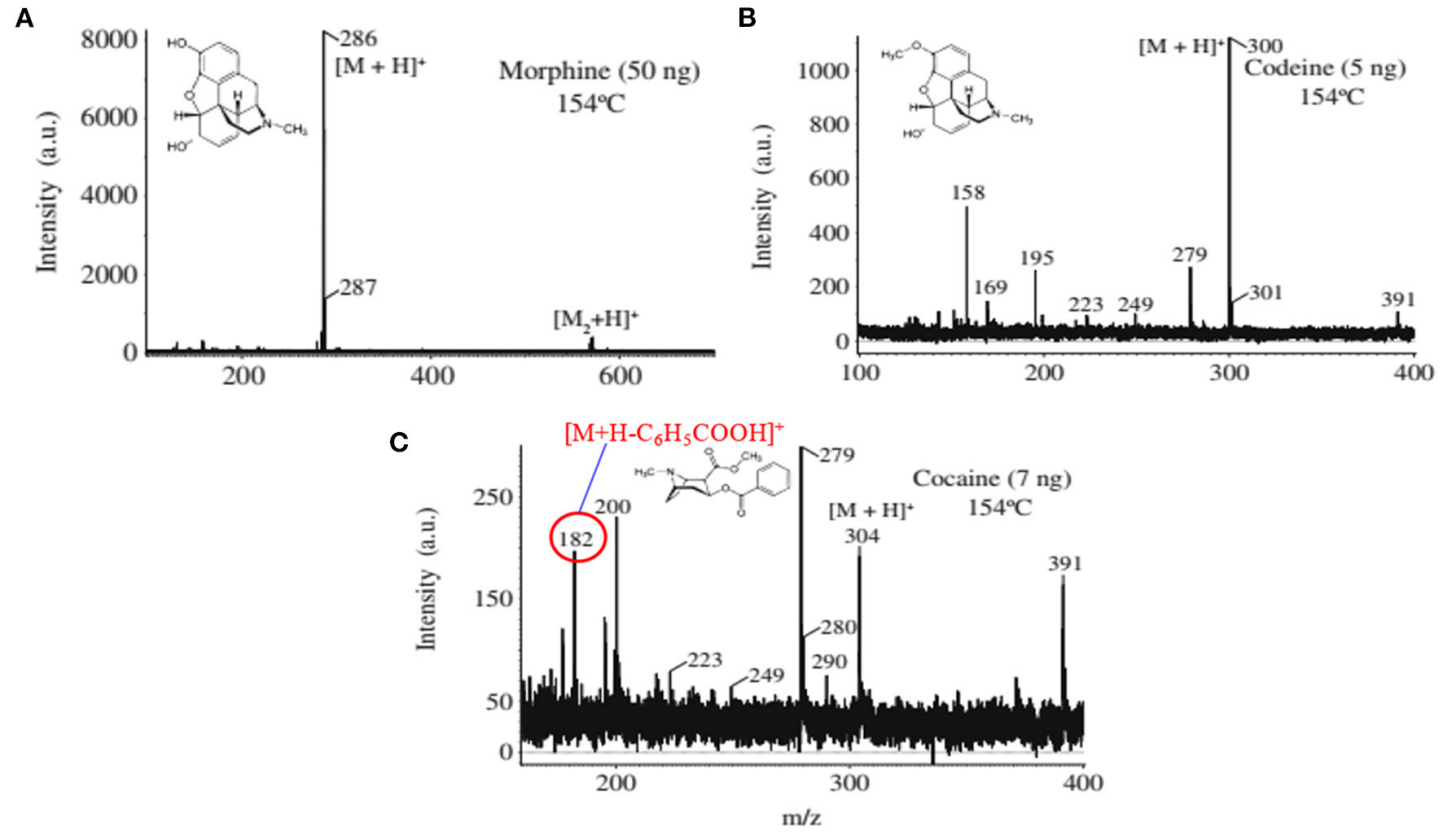
Mass spectra for the drugs of abuse **(A)** morphine, **(B)** codeine, and **(C)** cocaine. The ions at *m/z* 158, 195, and 279 in **(B)** are from background. Reproduced under permission from ACS (Usmanov et al., 2013).

As mentioned above, we found enhanced sensitivity for the relevant non-volatile drugs of abuse by using the ultrasonic cutter blade-based desorption method coupled with the same He-DBDI-MS system where the relevant analyte was deposited on the cutter blade instead of the PFA substrate and then rubbed (Figure 2 and Table 1) (Bi et al., 2021). The values of the limit of detections (LODs) for the morphine, cocaine and codeine were 40.38 ± 1.55, 20.00 ± 0.91, and 50.25 ± 1.85 pg, respectively, that were at least 8–10 time lower than that obtained for the cutter blade-based desorption method where the analyte compounds were deposited on the PFA substrate (Table 1). As described above, the enhanced sensitivity is due to synergistic effect caused by gaining the vibrational/oscillation and frictional/mechanical energy by the ultrasonic cutter when the analyte compounds deposited on the blade (Bi et al., 2021). By using the similar homemade He-DBDI and ion trap MS, Usmanov et al. (2013) reported quite high LOD values for morphine, cocaine and codeine by a heated filament (154°C) as a desorption means and these were 25.0, 7.0, and 3.0 ng, respectively (Usmanov et al., 2013). A brief description of this system has been elaborated in Figures 5, 6. A homemade ac-APCI ion source has also been fabricated by Usmanov and Hiraoka (2016) for characterization of morphine, cocaine and codeine and the LOD values were found to be 5.0, 5.0, 0.5 ng, respectively (Usmanov and Hiraoka, 2016). However, Jackson et al. (2010) reported only 15, 150 pg for cocaine and codeine, respectively where that for morphine was quite high, 3,000 pg, by using a heated substrate coupled to the DBDI-MS/MS system. The low LOD values for the cocaine and codeine using the tandem mass spectrometry (MS/MS) system is desirable, however, the high LOD value (3,000 pg) for morphine is suggesting the difficulties to analyze morphine at ultra-trace level using the heated substrate as a desorption process is challenging.

A heated filament-based desorption method has been used in order to desorb solid drug of abuse compounds and ionized/detected by a homemade He-DBDI coupled with a MS system. Such an experimental setup is shown in Figure 5 (Usmanov et al., 2013). Results show that a significant fragmentation for cocaine was observed using the heated filament-based desorption method (154°C) and the fragmented peak appeared as a protonated at *m/z* 182, [M+H-C_6_H_5_COOH]^+^ (Figure 6C) while morphine (Figure 6A) and/or codeine (Figure 6B) did not show any considerable fragmentation using the same desorption system at 154°C (Usmanov et al., 2013). However, morphine underwent fragmentation as the filament temperature increased from 154 to 200°C and appeared at *m/z* 268, [morphine-H_2_O+H]^+^ (Usmanov et al., 2013). They also found poor levels of LODs for the drugs of abuse using the heated filament-based desorption method coupled with the homemade He-DBDI-MS system (Table 1).

Therefore, it is concluded that coupling the cutter blade-based desorption method with the homemade He-DBDI-MS system will be a dignified analytical tool for detection of the non-volatile illicit drugs such as morphine, cocaine, codeine etc. at ultra-trace levels even from body fluids.

### Analysis of Explosives

**Figure 7:**
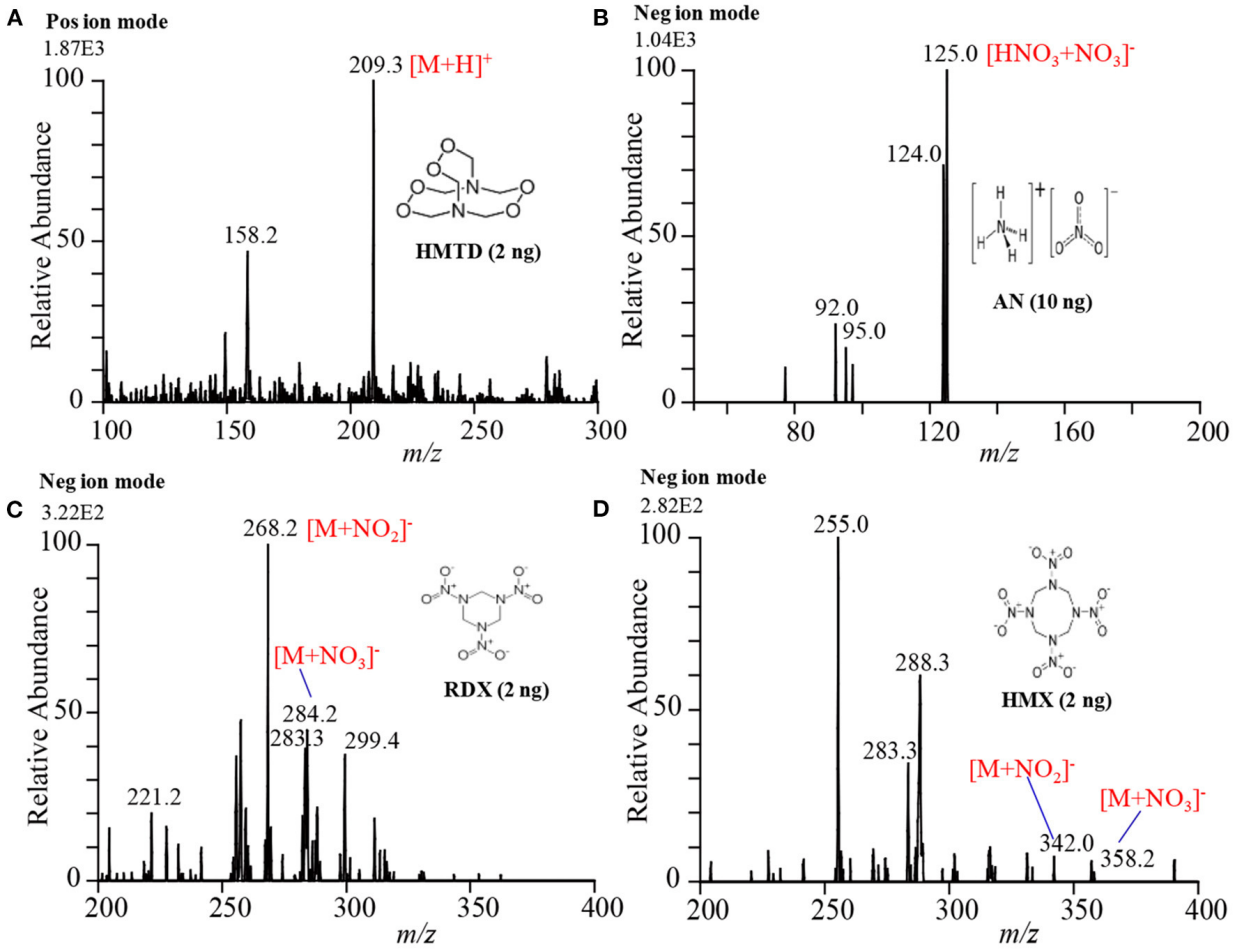
Mass spectra for **(A)** HMTD (2 ng), **(B)** AN (10 ng), **(C)** RDX (2 ng), and **(D)** HMX (2 ng). Each of the explosives was deposited on a perfluoroalkoxy (PFA) substrate and then rubbed by an ultrasonic cutter blade for desorption. The gaseous molecules were then ionized using a homemade helium DBDI ion source and detected by an iron trap MS. Reproduced under permission from ACS (Habib et al., 2014).

Exactly, 2 μL of the explosive compound of interest in acetonitrile solution was deposited on PFA substrate and dried in air. An ultrasonic cutter blade was used to desorb the deposited molecules into the gaseous phase by gentle touches with the PFA's surface. The gaseous analyte molecules were then ionized using a homemade He-DBDI and detected by an ion trap MS. Figure 7A shows the positive mass spectrum for 2 ng HMTD and Figures 7B–D show the negative mass spectra for 10 ng AN, 2 ng RDX, and 2 ng HMX, respectively. As seen from Figure 7A, HMTD gave the protonated ion, [M+H]^+^, that appeared at *m/z* 209. The origin of the peak that appeared at *m/z* 158 has been confirmed with and without laboratory hand gloves. It was confirmed that laboratory hand gloves is the origin of the peak that appeared at *m/z* 158 (Habib et al., 2013, 2014, 2015, 2020). The other peaks in Figure 7A were not identified.

In contrast, RDX and HMX were detected as adduct ions with ${\text{NO}}_{2}^{-}$ and ${\text{NO}}_{3}^{-}$ such as [RDX+NO_2_]^−^ (*m/z* 268), [HMX+NO_2_]^−^ (*m/z* 342), [RDX+NO_3_]^−^ (*m/z* 284), [HMX+NO_3_]^−^ (*m/z* 358) in the negative mode ionization as shown in Figures 7C,D, respectively. Ammonium nitrate (AN), however, was detected as a cluster ion of HNO_3_ with ${\text{NO}}_{3}^{-}$, [HNO_3_+NO_3_]^−^, (see Figure 7B). It is noted that the reactant ions ${\text{NO}}_{3}^{-}$ and ${\text{NO}}_{2}^{-}$ were the major background ions formed by the present ambient He-DBD ion source under the ambient conditions. The presence of the plenty of ${\text{NO}}_{2}^{-}$ and ${\text{NO}}_{3}^{-}$ ions was facilitating the formation of the adduct/cluster ions of RDX and HMX and also the adduct ion of HNO_3_ with the ${\text{NO}}_{3}^{-}$ for AN. Both the ${\text{NO}}_{2}^{-}$ and ${\text{NO}}_{3}^{-}$ ions have trigonal planar geometry and the oxygen atoms of each ion are highly negatively charged. The nitrogen atoms of the nitro (NO_2_) groups in the RDX and HMX molecules carry highly electropositive charge, thus they must form multidentate electrostatic bonds with the negatively ${\text{NO}}_{2}^{-}$ and ${\text{NO}}_{3}^{-}$ ions, thereby resulting in the formation of the cluster/adduct ions for RDX and HMX with these nitroxo anions (Usmanov et al., 2013; Habib et al., 2014; Bi et al., 2021). However, AN directly doesn't form any cluster ion with the ${\text{NO}}_{2}^{-}$ and/or ${\text{NO}}_{3}^{-}$ ion, rather it forms a cluster ion of HNO_3_ with the ${\text{NO}}_{3}^{-}$ ion. Ammonium nitrate dissociates into NH_3_ and HNO_3_ in the DBD plasma as follows: NH_4_NO_3_ NH_3_ + HNO_3_ (Usmanov et al., 2013; Habib et al., 2014). The formed HNO_3_ then forms cluster/adduct ion with the ${\text{NO}}_{3}^{-}$ ion, [HNO_3_+NO_3_]^−^, that appeared at *m/z* 125 (Usmanov et al., 2013; Habib et al., 2014; Bi et al., 2021).

It is suggested that the proton of the HNO_3_ molecule acts as a bridge between the two ${\text{NO}}_{3}^{-}$ ions. Analytical curves for the explosives of interest were made in order to evaluate the performance of the developed tribological desorption as well as the homemade He-DBDI methods. The values of limit of detection (LODs) are shown in Table 1. Other ambient ionization methods such as DESI (Takáts et al., 2005), EESI (Chen et al., 2009), DBDI (Zhang et al., 2009; Garcia-Reyes et al., 2011) have also been used for the detection and quantification of explosive compounds. It seems that the developed ultrasonic cutter-based tribodesorption (TD) method can work for efficient non-thermal desorption of the non-volatile drugs of abuse as well as explosives. The coupling of the tribodesorption with DBDI must have potentiality for trace-level detection of non-volatile drugs of abuse and explosives from analytical point of view.

Among the MS systems, ultrasonic cutter blade-based desorption process coupled with the He-DBDI-MS system exhibited as a promising analytical tool for analysis of highly non-volatile explosive compounds at ultra-trace levels (Habib et al., 2014; Bi et al., 2021). Further attempts have already been taken to fabricate a commercial hybrid MS system through combination of the ultrasonic cutter blade, He-DBD ion source and an ion trap MS for analysis of highly non-volatile compounds such as drugs of abuse, explosives etc. at ultra-trace levels.

## Headspace-Dielectrictric Barrier Discharge (DBD) Ionization-MS System

Amphetamines belong to an amine group under the aliphatic compounds and they can significantly stimulate the central nervous system. Of them, amphetamine (AM) and methamphetamine (MA) and their methylenedioxy derivatives such as 3,4-methylenedioxyamphetamine (MDA) and 3,4-methylenedioxy methamphetamine (MDMA) are the main amphetaminic compounds that have been commonly used by athletes, drug addicts and recreational users as stimulants (Perez-Reyes et al., 1991; Ropero-Miller and Goldberger, 1998). Therefore, rapid detection of illicit drugs such as amphetaminic compounds in urine is very much needed in order to examine the consumption of these illicit drugs by the athletes as well as the young population in developing countries like Bangladesh, Myanmar, India, Thailand, China etc.

**Figure 8:**
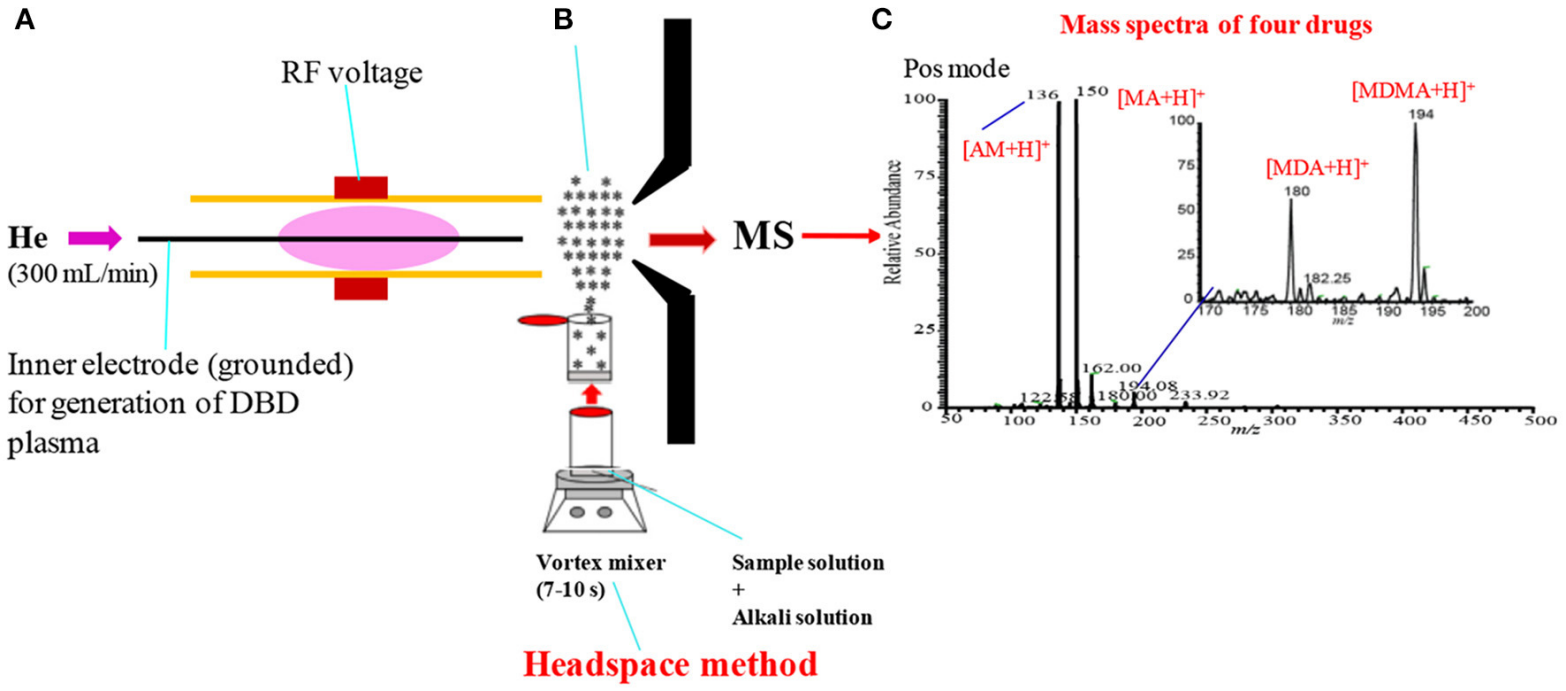
A schematic of headspace-He-DBDI coupled with MS **(A)**, headspace method **(B)** and mass spectrum of four amphetaminic drug compounds in positive ion mode **(C)**. In headspace method, the drugs of abuse of interest were treated with alkali solution for gasification. Reproduced under permission from Elsevier (Habib et al., 2020).

Figure 8 shows an experimental setup of the headspace-He-DBDI coupled with MS system for gasification of amphetaminic drugs of abuse through headspace method. The presence of amine and/imine groups in the amphetaminic compounds causes their volatility, thus these compounds treat with hydrochloric acid (HCl) in order to provide their stability. The addition of hydrochloric acid causes the formation of the quaternary cationic nitrogen centered where chloride (Cl^−^) acts as a counter ion, thereby resulting in gaining stability of the amphetaminic compounds. Thus, alkali treatment of the quaternary amphetaminic compounds ceases the free base amphetaminic compounds into gaseous phase that is called as a headspace method. In the present section, the headspace method was applied for the gasification of the amphetaminic compounds spiked raw urine. Herein, MA, MA, 3,4-methylenedioxyamphetamine (MDA) and 3,4-methylenedioxymethamphetamine (MDMA) were taken as model compounds to evaluate the analytical performance of the headspace-DBDI-MS system. These amphetaminic compounds were spiked in raw urine and treated with non-carbonated (e.g., NaOH, KOH etc.) and carbonated (Na_2_CO_3_, K_2_CO_3_ etc.) alkali solutions (Habib et al., 2020). Figure 5B shows such an example of headspace method. Figure 5C shows positive headspace-DBDI mass spectra for a mixture of AM, MA, MDA, and MDMA in aqueous solution. As seen from Figure 5C, both the AM and MA show their protonated ions, [AM+H]^+^, [MA+H]^+^, as the major ions, and MDA and MDMA also provide their protonated ions, [MDA+H]^+^, [MDMA+H]^+^, but with much weaker ion signals. This is because of the difference in vapor pressures of the amphetamic compounds as described in the following section (Habib et al., 2020).

As mentioned above, amphetamine compounds are aliphatic amines, thus they are either volatile and/or semi-volatile in nature. According to the literature, the vapor pressures of AM, MA, MDA, and MDMA are 310.0 × 10^−3^, 5.4 × 10^−3^, 1.0 × 10^−3^, and 1.6 × 10^−3^ mm of Hg at 25°C, respectively (Habib et al., 2020). Amphetamine compounds are mainly aliphatic amines thus, they have positive proton affinity. Accordingly, they convert into quaternary amines just mixing with hydrochloric acid where chloride (Cl^−^) ion acts as a counter ion. Formation of the quaternary amines prevents the volatility of the amphetamines that provides their better stability. Addition of alkali solution to the quaternary amines evolves gaseous free base amines because of their considerable vapor pressures at room temperature. The homemade He-DBDI seems as an atmospheric pressure chemical ionization (APCI) because of formation of hydronium ion, H_3_O^+^, in the positive mode of operation (Habib et al., 2014, 2020). In addition, the proton affinity (PA) of this group of compounds is quite high i.e., PA for MA is 965 kJ/mol, and follows the increasing order: AM < MDA < MA < MDMA (Matsumura et al., 2003). Thus, these compounds efficiently form their protonated molecular ion, [M+H]^+^, in the positive ion mode.

**Figure 9:**
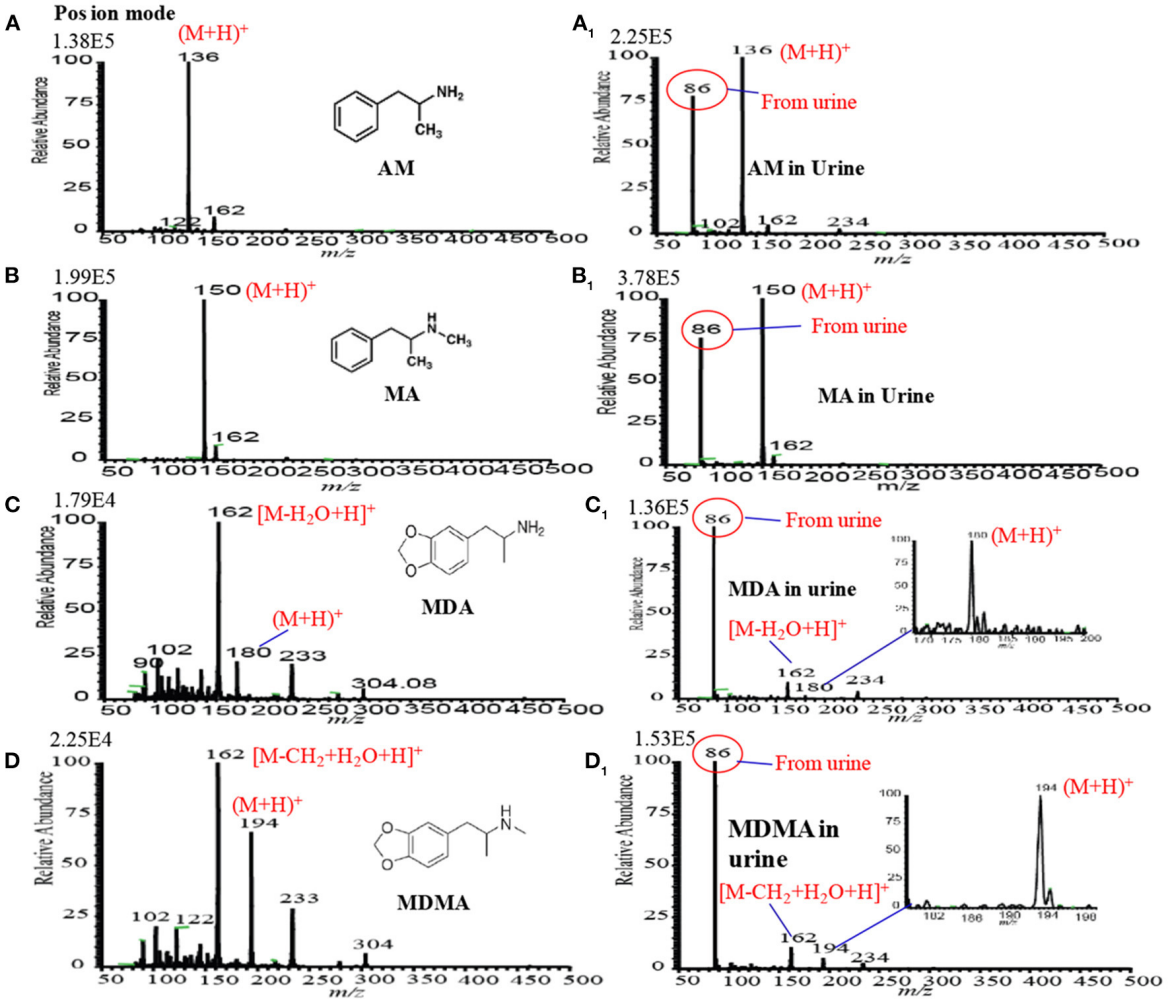
Mass spectra for **(A)** amphetamine (AM), **(A1)** amphetamine in urine, **(B)** methamphetamine (MA), **(B1)** methamphetamine (MA) in urine, **(C)** 3,4-methylenedioxyamphetamine (MDA), **(C1)** 3,4-methylenedioxyamphetamine (MDA), **(D)** 3,4-methylenedioxymethamphetamine (MDMA) and **(D1) (D)** 3,4-methylenedioxymethamphetamine (MDMA) measured by headspace-DBDI-MS system. Amount of each amphetamine compound was 1 ng/mL in water as standard and spiked in raw urine and then treated by equal volume of ammoniated K_2_CO_3_ solution [85% K_2_CO_3_ (4 M) + 15% NH_3_ (28%)]. Reproduced under permission from Elsevier (Habib et al., 2014).

Figures 9A–D_1_ show the positive headspace-DBDI mass spectra for AM, MA, MDA, and MDMA, respectively. The values at the top-left corner in each spectrum indicate the intensities of the ions of interest. Figures 9A,A_1_ show the positive mass spectra for AM in water and spiked raw urine, respectively. As seen from Figures 9A,A_1_, the ion intensity for the [AM+H]^+^ ion is relatively higher for AM spiked urine than that for water, however, the presence of urea, creatinine in raw urine suppress the ion signals of the analytes of interest (Habib et al., 2020). Similar observation was also observed for MA (see Figures 9B,B_1_), MDA (see Figures 9C,C_1_) and MDMA (see Figures 9D,D_1_). The volatile components of the raw urine (e.g., urea, creatinine etc.) play crucial roles for the better sensitivity of these amphetaminic compounds. Addition of NaOH and/or KOH to the solution of the relevant compound, the quaternary nitrogen converts to free base amine where the volatile urine components act as carrier gas to bring the free base amine to the gas phase. However, urine's components must cause ion suppression of the analytes of interest. In this study, non-carbonated and carbonated alkali solutions were used for the ceasing amphetaminic compounds in the headspace method (Habib et al., 2020).

It is noted that carbonated alkali solution enhances the sensitivity for these amphetaminic compounds. The quaternary nitrogen containing amine compounds are acidic in nature, thus, a reaction might occur between the acidic amphetaminic compounds and the alkali Na_2_CO_3_ or K_2_CO_3_, thereby resulting in the formation of gaseous CO_2_ as well as free base amines. The generation of gaseous CO_2_ in *in-situ* acts as a carrier gas for the free base amines. A trace amount of ammonia solution with the alkali solution also causes better sensitivity for the amphetaminic compounds. To find the quantitative capabilities of the headspace-DBDI-MS system, analytical validation such as LOD, precision, linear range correlation coefficient (*R*^2^) and analyte recovery rate were investigated with three replicates (*n* = 3). Peak intensities, with the signal-to-noise ratio (S/N) of at least 3, the protonated molecular ion, [M+H]^+^, of the analyte of interest were used to construct the analytical calibration curves. Each of the points in the calibration curve was at least three in replication. The minimum amount of the calibration curve has been considered as the LOD of the analyte of interest. It is noteworthy to mention that amphetamine compounds spiked in raw urine exhibited better LOD compared to the corresponding standard solutions. The values of LOD for the AM, MA, MDA, and MDMA from standard and spiked in urine are tabulated in Table 1 (Habib et al., 2020). Therefore, it is concluded that the developed headspace-DBDI system can be applied for trace level detection of amphetaminic compounds even from biological samples like urine, but the method may not be suitable for the non-volatile drugs of abuse such as morphine, cocaine, codeine, heroin etc.

In summary, the presence of amine and/or imine groups in the amphetaminic compounds makes them suitable for the headspace method in order to desorb into the gaseous phase which is the mandatory step for ionization in MS. On the other hand, morphine, cocaine, codeine, heroin etc. are rather non-volatile compounds and they do not have any amine and/or imine groups, thus these drugs of abuse compounds need mechanical and/or thermal desorption method rather than chemical treatment as described in the preceding section.

## Hollow Cathode Discharge (HCD) Ionization-MS System

**Figure 10:**
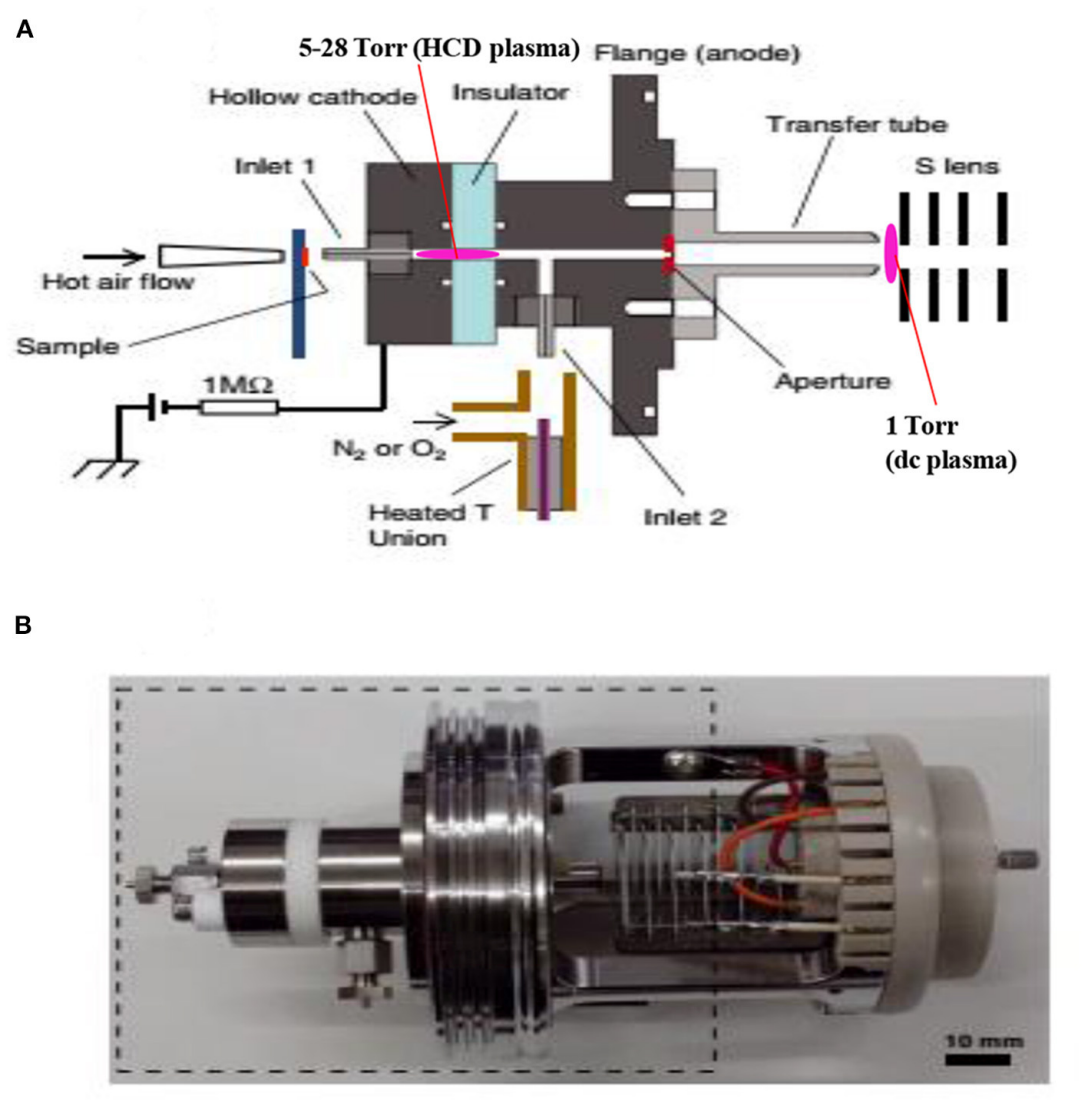
A schematic of the fabricated hollow cathode discharge (HCD) ion source **(A)**. The length of cathode electrode is 5 mm and its inner diameter is 2 mm. An insulator made of aluminum oxide (5 mm thick) is for separation of the cathode and anode. An aperture with 1 mm diameter is for getting ions into the 1 Torr vacuum stage is 1 mm. A stainless steel capillary (ion transfer tube) with an i.d. of 4 mm is used to transfer ions from the ion source to the S-lens. The difference between the aperture and the exit of the transfer tube is 24 mm. There is a ~3 mm gap between the edge of the transfer tube and the first electrode of the S-lens. A photograph for the HCD ion source **(B)**. The dotted-line box around the portion of the photograph shows part **(A)**. Reproduced under permission from Wiley (Habib et al., 2015).

In mass spectrometry, ion source plays a vital role for detection and quantification of analytes of interest at trace to ultra-trace levels. Hence, much attention has been paid to fabricate new ion sources for MS that can efficiently ionize the compounds of interest, thereby resulting in realizing ultra-trace level detection. Herein, a hollow cathode discharge (HCD) ion source has been fabricated for the detection of mainly explosives. A schematic of the HCD ion source is shown in Figure 10A where Figure 10B stands for its photograph. The salient feature of the developed HCD ion source is air its carrier gas instead of rare gases like He or Ar, thus HCDI-MS system can deploy at public places such as airports, railway stations, cultural sites etc. for characterization of explosives. The use of air as a carrier gas in the HCD ion source can also ensure no need for periodic change of reagent gas. The HCD ion source was kept as turned on condition at 5 Torr using air as a carrier gas for a month in order to evaluate its robustness and found no severe corrosion inside of the electrodes or insulators with naked eye.

As seen from Figure 10A, the ion source has two inlets for introducing samples under ambient conditions. The inlets made of stainless steel capillary with the length and inner diameter (i.d.) are 10 and 0.25 mm, respectively. The HCD ion source consists of two metallic electrodes made of stainless steel. The two electrodes are separated by an insulator made of ceramic with 5 mm thick. The inlet-1 is connected to the cathode while inlet-2 is connected to the flange of the ion source that acts as an anode (Figure 10A). Inlet-1 is used for ambient sampling throughout the experiments while the inlet-2 closed. A high-voltage power supply (Pulse Electronic Engineering Co. Ltd, Chiba, Japan) was connected to the cathode (length: 2 mm; i.d.: 2 mm) while the flange of the ion source (stainless steel) that acted as an anode was kept at ground potential.

According to the design of the HCD ion source, the ion source pressure can be varied using an aperture of 1 mm diameter by placing it between the flange and the transfer tube (Figure 10A). The gas pressure in the HCD ion source was measured through installing a pressure gauge (SMC, Tokyo, Japan) at the port of inlet-2. The pressure gauge showed a gas pressure of 28 Torr by placing the aperture between the flange and the transfer tube while that was 5 Torr when the aperture was removed. The flow rate of air through the inlet-1 was calculated in order to know an empirical flow rate of the analyte of interest and the calculated flow rate was found to be ~360 mL/min. The ions formed in the HCD ion source were transferred using a transfer tube made of stainless steel to the S-lens of a linear ion trap mass spectrometer (LTQ XL, Thermo Scientific, San Jose, CA, USA). A rotary vane pump was used to keep the pressure at 1 Torr in the first pumping stage in the MS. It is noted that an ion trap mass spectrometer requires high vacuum conditions in order to run the MS properly.

A high-voltage power was applied to the cathode to generate HCD plasma. The potential was just above the threshold of the gaseous breakdown voltage to stabilize the HCD glow. The potential and current were kept constant at −1.30 kV (0.3 mA) and −1.62 kV (0.3 mA) for the ion source pressures 5 and 28 Torr, respectively. Herein, the discharge current was kept relatively at low in order to facilitate soft ionization of the labile explosives while Ganeev et al. (2007) reported that the HCD ion source can be operated within a wide range from a few mA to several hundreds of mA (Ganeev et al., 2007).

As seen from Figure 10A, a dc plasma was generated between the transfer tube and the S-lens by applying +0.85 kV (0.12 mA) and −0.85 kV (0.15 mA) to the transfer tube in the positive and negative ion modes, respectively under 1 Torr ion source pressure in order to investigate the ionization mechanism of the relevant explosives with ion source pressure. A linear ion trap mass spectrometer was used to monitor the ions generated by the HCD ion source for the explosives of interest through adjusting the instrumental settings as follows: 150–200°C for ion transport tube; 80% for S-lens and 600–800 V for RF ion guide voltage. The auto gain control was enabled with a maximum ion injection time of 2 ms, and the number of micro scans was 1. A tandem mass spectrometry (MS/MS) mode was used for characterization of the ions formed from the explosive compounds. Herein, the collision-induced dissociation (CID) mode with the collision energy within a range from 20 to 50% was used where helium was used for collision (Habib et al., 2015).

A microscopic glass slide was used as a sample holder. Exactly 2 μL solution of the relevant explosive was deposited on the slide and kept for air drying. The sample spot diameter was ~2 mm. An air heater (ranging from 150 to 225°C) attached with a quartz nozzle (i.d. 2 mm) was used to vaporize the solid compounds through heating from the backside of the sample spot. A thermocouple was used to observe the heater temperature. The solid explosives were gasified by using the hot air except TATP. This explosive was kept in a glass vial like a headspace because of its high vapor pressure at room temperature and then introduced into the ion source for ionization through the inlet-1 (Figure 10A).

To evaluate the quantitative capabilities of the fabricated HCD ion source, analytical validation, for example, limit of detection (LOD), precision and linear range of coefficient of determination (*R*^2^) were investigated with at least four replications (*n* = 4). Peak areas for the relevant ions, as shown underlined in Table 1, were used to construct the analytical calibration curves for HMTD, RDX, PETN, and TNT. The values of the LOD for the explosive compounds of interest were based on a signal-to-noise ratio (S/N) of at least 3 (Habib et al., 2015). The constructed calibration curves exhibited about two orders of magnitude of their linear range with reproducible signal intensities.

The hollow cathode discharge (HCD) ion source has been fabricated in order to analyze the explosive compounds as shown in Table 2. Mass spectra for TATP and HMTD in the positive mode and that for RDX, PETN, TNT, and NG in the negative mode were measured through adjusting the ion source pressures at 1, 5, and 28 Torr. The ion source temperatures for analysis of the explosives are also tabulated in Table 2 (Habib et al., 2015).

**Table 2:** Limits of detection (LODs) (pg) and observed ions (m/z values in parentheses) for the explosives examined at three ion source pressures.

**Ions observed at three ion source pressures with** ***m/z*** **values in the ion source parentheses**					**LOD (pg) at three ion source pressures** **(Torr)**		
**Compounds** **(molecular mass)**	**Temperature** **(^**°**^C)**	**1 Torr**	**5 Torr**	**28 Torr**	**1**	**5**	**28**
TATP (222)	150		[C_3_H_6_O_2_+H]^+^ (75) **[C**_**3**_**H**_**6**_**O_3_+H]**^**+**^ **(91)**	[C_3_H_6_O_2_+H]^+^ (75)**[C**_**3**_**H**_**6**_**O_3_+H]**^**+**^ **(91**)[M+NH_4_]^+^ (240)	-	-	-
HMTD (208)	180		**[M+H]**^**+**^ **(209)** [M–CH_2_O]^+^ (179)	[M**+H]**^**+**^**(209)** [M–CH_2_O]^+^ (179)	-	100	20
TNT (227)	150	**M**^**−**^(227)[M-NO]^−^ (197)[M-OH]^−^ (210)	M^−^ (227) **[M-H]**^**−**^ **(226)** [M-NO]^−^ (197) [M-2NO]^−^ (167) [M-3NO]^−^ (137) [M-H+O]^−^ (242)	[M-H]^−^ (226)[M+H]^−^ (228)**[M-H+O]**^**−**^ **(242)**	2	50	70
NG (227)	150			[M**+NO**_**3**_**]**^**−**^**289**	-	-	300
PETN (316)	180	**[M–CH**_**2**_**ONO**_**2**_**]**^**−**^ **(240)**	**[M–CH**_**2**_**ONO**_**2**_**]**^**−**^ **(240)** [M+NO_3_]^−^ (378)	[M+NO_2_]^−^ (362) [M**+NO**_**3**_**]**^**−**^**(378)**	1000	1000	800
RDX (222)	180	[M–NO_2_-CH_2_NNO_2_]^−^ (102)**[M–NO**_**2**_**-HNO**_**2**_**]**^**−**^ **(129)**[M–NO_2_]^−^ (176)	**[M–NO**_**2**_**-HNO**_**2**_**]**^**−**^ **(129)** [M+NO_2_]^−^ (268) [M+NO_3_]^−^ (284)	[M–NO_2_-HNO_2_]^−^ (129) [M+NO_2_]^−^ (268) [M**+NO**_**3**_**]**^**−**^**(284)**		30	40

*Bold type indicates the major ion. The ions used to obtain analytical curves are underlined*.

*Reproduced under permission from Wiley (Habib et al., 2015)*.

**Figure 11:**
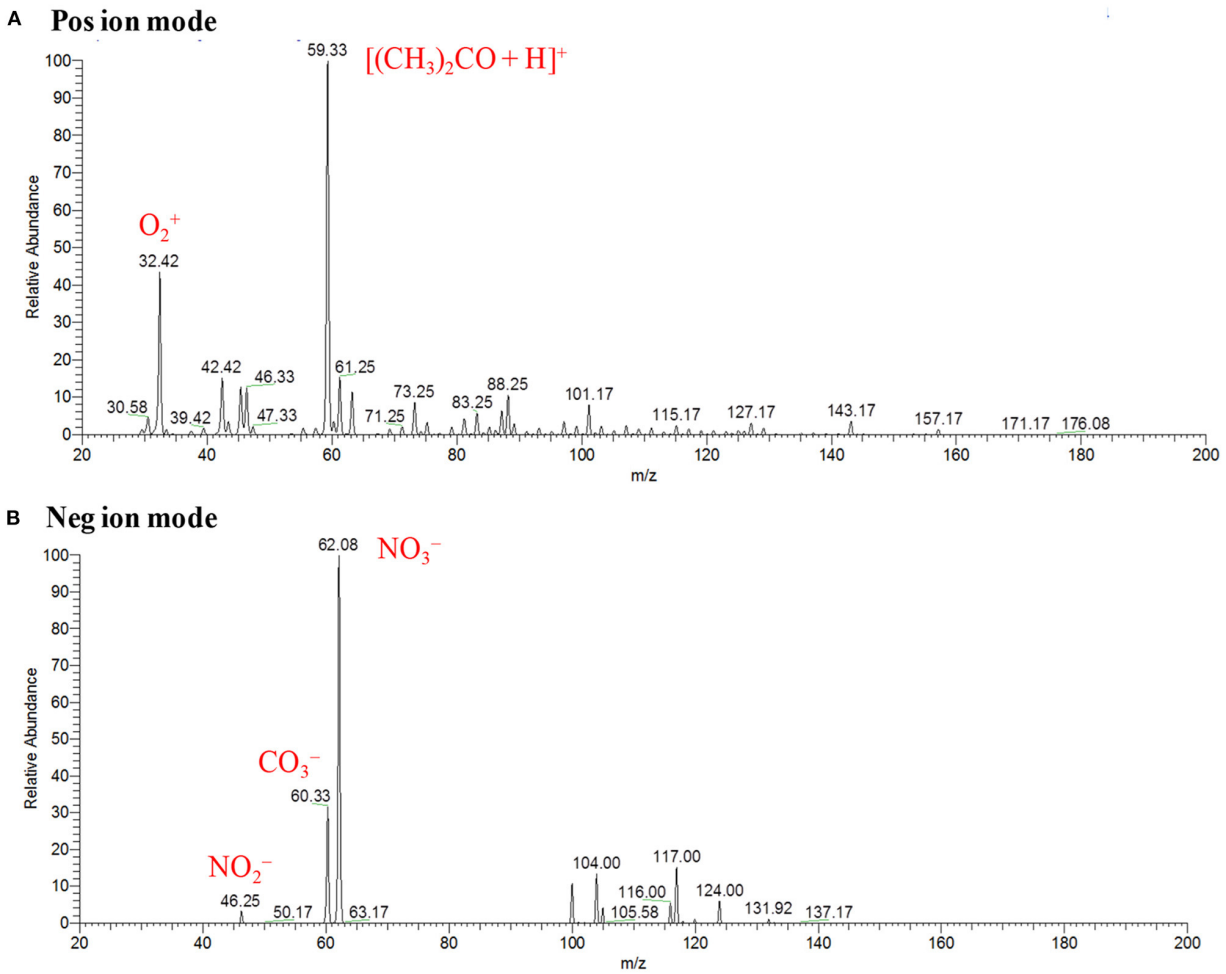
Background mass spectra for hollow cathode discharge (HCD) ion source measured at 5 Torr: **(A)** positive ion mode and **(B)** negative ion mode. Reproduced under permission from Wiley (Habib et al., 2015).

Figures 11A,B show the background mass spectra for the HCD ion source recorded at 5 Torr in the positive and negative ion modes, respectively. As seen from Figures 11A,B, oxygen molecular ion, ${\text{O}}_{2}^{+}$ (*m/z* 32), and protonated acetone, [(CH_3_)_2_CO+H]^+^ (*m/z* 59), are the major ions in the positive ion mode while ${\text{NO}}_{2}^{-}$ (*m/z* 46), ${\text{CO}}_{3}^{-}$ (*m/z* 60), and ${\text{NO}}_{3}^{-}$ (*m/z* 62) ions are observed as the major ions in the negative mode of operation. In the positive ion mode, protonated acetone, [(CH_3_)_2_CO+H]^+^ (*m/z* 59), as the base peak is reasonable. This is because laboratory air is commonly contaminated with organic solvent such as acetone, ethanol, methanol, ammonia etc. The formation of the protonated acetone, [(CH_3_)_2_CO+H]^+^, ion acts as the major regent ion for the protonation of TATP and HMTD in the positive ion mode. The ambient air molecules became excited by the HCD plasma that causes the formation of the ${\text{NO}}_{2}^{-}$, ${\text{CO}}_{3}^{-}$ and ${\text{NO}}_{3}^{-}$ ions and appeared in the negative ion mode as shown in Figure 11B. Sekimoto and Takayama (2010) described the mechanism of formation of the ${\text{NO}}_{2}^{-}$, ${\text{CO}}_{3}^{-}$ and ${\text{NO}}_{3}^{-}$ ions in the plasma-excited air as well (Sekimoto and Takayama, 2010). Other background signals in Figure 11 were not identified.

In conclusion, the fabricated HCD ion source was applied to analyze explosives such as TATP, HMTD, RDX, PETN, TNT, and NG at different ion source pressures in order to investigate ion formation mechanisms. The formation of protonated acetone, [(CH_3_)_2_CO+H]^+^, as a reagent ion in the positive ion mode facilitates the formation of the protonated compounds of interest such as [HMTD+H]^+^, [TATP+H]^+^, and its protonated fragment ion, [C_3_H_6_O_3_+H]^+^ (*m/z* 91) etc. while the formation of ${\text{NO}}_{2}^{-}$, ${\text{NO}}_{3}^{-}$ in the negative ion mode causes the formation of cluster ions of RDX, PETN and also dehydrogenated of TNT, [TNT-H]^−^ (*m/z* 226) etc.

### Mass Spectra of Explosives by HCD Ion Source in Positive Ion Mode

**Figure 12:**
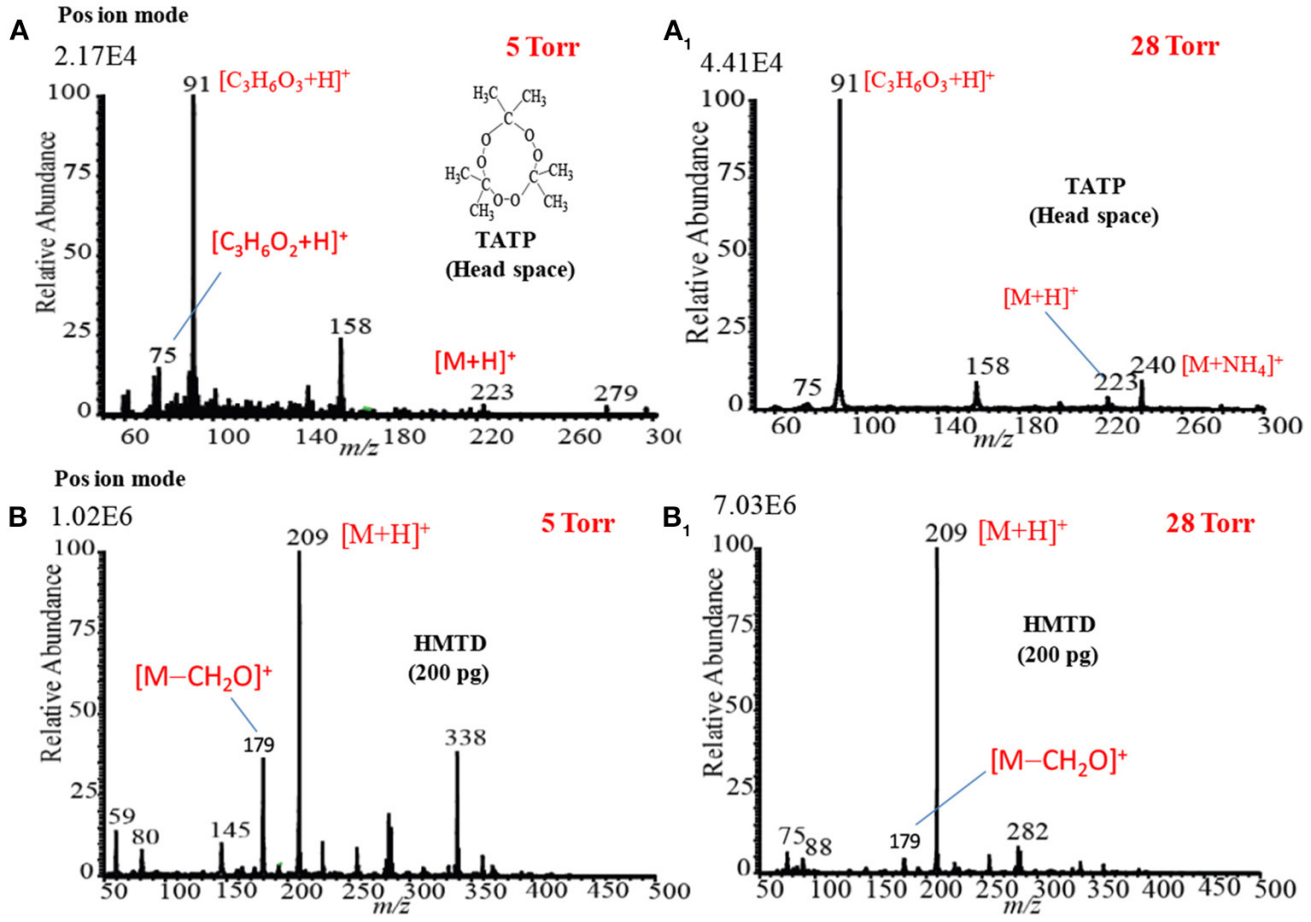
Positive ion mode hollow cathode discharge (HCD) mass spectra for **(A)** TATP (headspace) measured at 5 Torr, **(A1)** TATP (headspace) measured at 28 Torr, **(B)** HMTD (200 pg) measured at 5 Torr, and **(B1)** HMTD (200 pg) measured at 28 Torr. Reproduced under permission from Wiley (Habib et al., 2015).

Figures 12A,A_1_ show the mass spectra for the headspace TATP measured by the HCD ion source in the positive ion mode at 5 and 28 Torr ion source pressure, respectively. As seen from Figures 12A,A_1_, TATP showed the protonated fragment ion, [C_3_H_6_O_3_+H]^+^, that appeared at *m/z* 91 as the major ion along with another protonated fragment ion, [C_3_H_6_O_2_+H]^+^ (*m/z* 75) and protonated molecular ion, [TATP+H]^+^ (*m/z* 223) as the minor ions, however, a new peak for the ammoniated TATP, [TATP+NH_4_]^+^, was observed at *m/z* 240 when the ion source pressure was kept at 28 Torr. In a blank experiment by introduction of methanol to the ion source, however, there were no ions observed at *m/z* 91 and 75. By using the ac/dc-APCI, we also found the ammoniated TATP, [TATP+NH_4_]^+^ (*m/z* 240), ion in the positive mode of operation (Habib et al., 2013). This is because of the contamination of laboratory air by the ammonia used in chemical laboratories for different purposes. The presence of ammonia in the laboratory air causes the formation of the adduct ion of the TATP with ammonia, [TATP+NH_4_]^+^ (*m/z* 240). The appearance of the adduct ion at 28 Torr is logical because of presence of requisite level of ammonia at high ion source pressure, 28 Torr, that facilitates the formation of the [TATP+NH_4_]^+^ ion. The formation of the adduct ion passes through its transition state complex, [TATP•••NH_4_]^+*^ and according to transition state theory, a third-body collusion is required to stabilize the intermediate transition state in order to obtain the product, [TATP+NH_4_]^+^ ion that can be ascribed by the following equation (Laidler, 1987):

(1) $$\text{TATP}+{\text{NH}}_{4}^{+}⇆{\left[\text{TATP}•••{\text{NH}}_{4}\right]}^{+*}\to {\left[{\text{TATP}+\text{NH}}_{4}\right]}^{+}$$

where T_B_ represents the third body.

Peak intensities of the relevant ion signals are shown at the upper left hand corner of the spectra. As shown in Figure 12A_1_, the intensities for the ions appeared at *m/z* 91, 223 were enhanced by a factor 2 compared to that as shown in Figure 12A. It is noted that high ion source pressure assists the formation of protonated acetone, [(CH_3_)_2_CO+H]^+^ (*m/z* 59) and/ or hydronium ion, H_3_O^+^, as reagent ions in the positive ion mode. The relatively high level of the reagent ions, [(CH_3_)_2_CO+H]^+^/H_3_O^+^, at high ion source pressure enhances the formation of the protonated ion of the relevant compounds. Therefore, it is concluded that plasma-based ionization methods facilitate the formation of protonated fragment ion, [C_3_H_6_O_3_+H]^+^, of the TATP that appeared at *m/z* 91. Takada et al. (2012b) also found the protonated fragment ion for TATP, [C_3_H_6_O_3_+H]^+^ (*m/z* 91), by using counter-?ow dc corona APCI (Takada et al., 2012b), however, electrospray-based ionization methods such as DESI (Cotte-Rodríguez et al., 2008), extractive electrospray ionization (EESI) (Chen et al., 2009) facilitate the formation of protonated, sodiated, and ammoniated TATP. Sigman et al. (2006) analyzed TATP by the chemical ionization, and performed a theoretical calculation using the density functional theory (DFT) in order to investigate the fragmentation pathway of the [TATP+H]^+^ (*m/z* 223) ion (Sigman et al., 2006). They also reported the formation of the protonated fragment ion for the TATP, [C_3_H_6_O_3_+H]^+^ (*m/z* 91), as well.

Figures 12B,B_1_ show the mass spectra of HMTD measured by HCD ion source at 5 and 28 Torr in the positive ion mode, respectively. Results show that HMTD appeared as its protonated molecular ion, [HMTD+H]^+^ (*m/z* 209), as the major with a protonated fragment ion, [HMTD+H-CH_2_O]^+^, as a minor ion that appeared at *m/z* 179 (Habib et al., 2015). It has also been reported that HMTD detected as its protonated form, [HMTD+H]^+^, by using discharge-based ion sources (Chen et al., 2010; Hiraoka et al., 2010; Garcia-Reyes et al., 2011; Habib et al., 2014). As seen from Figure 12B_1_, intensity of the protonated molecular ion, [HMTD+H]^+^, increased by a factor of 7 as the ion source pressure increases from 5 to 28 Torr and the ratio of [HMTD-CH_2_O]^+^/[HMTD+H]^+^ decreased. Hiraoka (2013) also reported that the higher ion source pressure stabilizes of a nascent protonated molecular ion by a third-body collision. It is noted that electrons generated in the HCD plasma gain relatively low kinetic energy under high gas pressure (Mavrodineanu, 1984). Therefore, it is concluded that high ion source pressure facilitates the detection of HMTD as its protonated form. The other peaks shown in Figures 12B,B_1_ were not assigned.

The high ion source pressure in the HCD plasma facilitates the formation of protonated molecular and/or protonated fragment ion in the positive ion mode. This is the reason for finding the enhanced intensity for the protonated ions at higher ion source pressure for HMTD and TATP. In conclusion, the high gas pressure is desirable in the positive HCD ion source in order to detect the explosive compounds at trace levels.

### Mass Spectra of Explosives by HCD Ion Source in Negative Ion Mode

**Figure 13:**
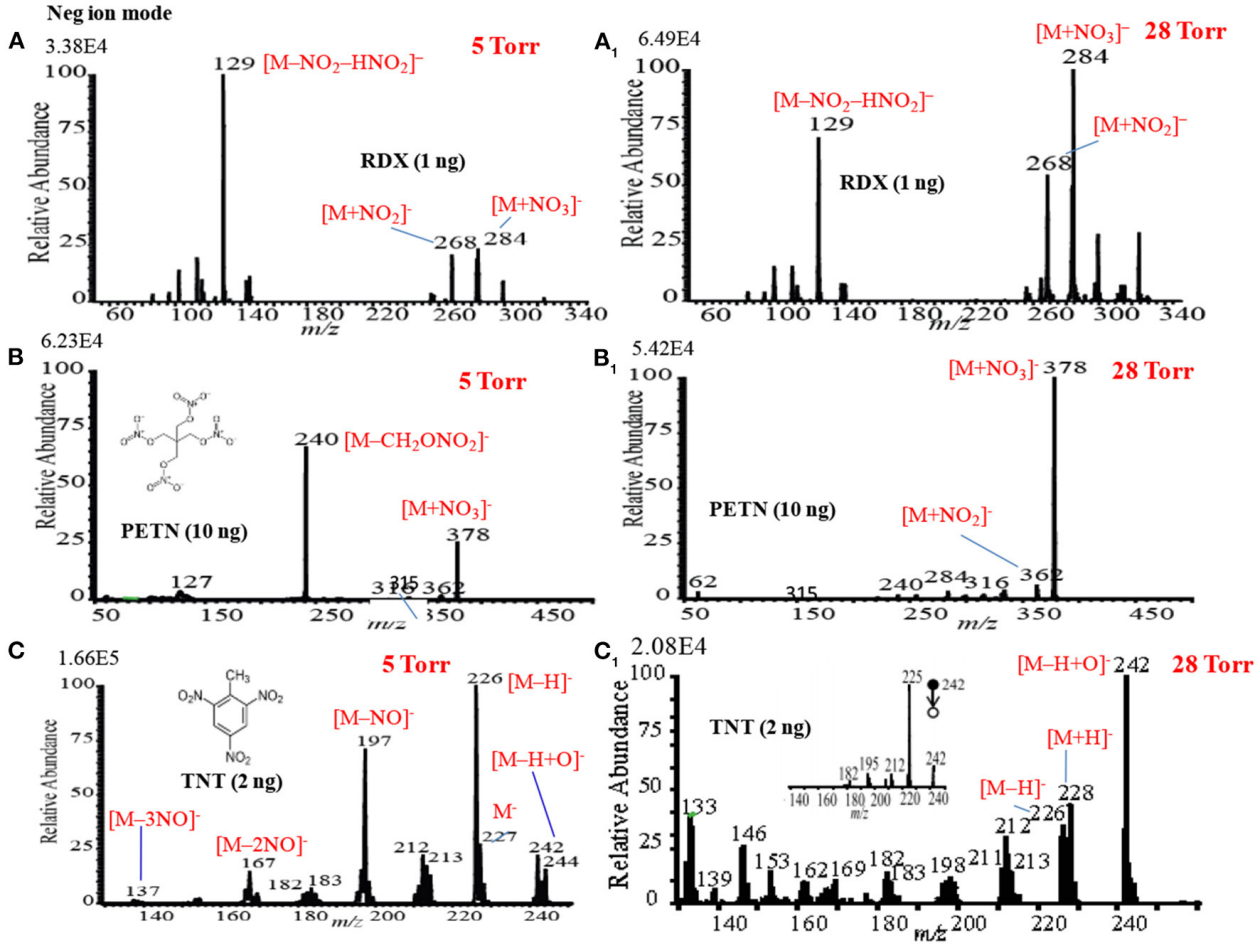
Mass spectra for **(A)** RDX (5 Torr), **(A**_**1**_**)** RDX (28 Torr), **(B)** PETN (5 Torr), **(B**_**1**_**)** PETN (28 Torr), **(C)** TNT (5 Torr), and **(C**_**1**_**)** TNT (28 Torr) measured by the HCD ion source in the negative ion mode. The MS/MS spectrum of [TNT-H+O]^−^ (*m/z* 242) is shown in the inset (CID: 25%). Reproduced under permission from Wiley (Habib et al., 2015).

Figures 13A,A_1_ show the negative mode mass spectra for 1 ng RDX measured by the HCD ion source at 5 and 28 Torr ion source pressure, respectively. As seen from Figure 13A, RDX exhibited severe fragmentation at 5 Torr, thus its fragment ion, [RDX-NO_2_-HNO_2_]^−^ (*m/z* 129), appeared as a base peak while its adduct ions, [RDX+NO_2_]^−^ (*m/z* 268) and [RDX+NO_3_]^−^ (*m/z* 284), appeared as minor ion signals. Contrary to the higher ion source pressure (28 Torr), the adduct ion of RDX with the reagent ion ${\text{NO}}_{3}^{-}$, [RDX+NO_3_]^−^ (*m/z* 284), appeared as a major ion where the ion intensity of the [RDX+NO_3_]^−^ ion was 8 times higher than that for 5 Torr. It is reasonable that the higher ion source pressure enhances the formation of the adduct ion of RDX, [RDX+NO_3_]^−^ (*m/z* 284). This is because the rate of formation of the reagent ions i.e., ${\text{NO}}_{2}^{-}$ and ${\text{NO}}_{3}^{-}$, in the negative ion mode increases with the ion source pressure. The presence of plenty of the reagent ions under the higher gas pressure acts as a third body that enhances the collisional probability between the RDX and ${\text{NO}}_{3}^{-}$, thus the transition state complex of RDX with ${\text{NO}}_{3}^{-}$, [RDX•••NO_3_]^−*^ gains adequate stabilization energy. By dc glow discharge ionization, RDX gave mostly fragment ions under 0.8 Torr of air pressure (McLuckey et al., 1996), however, adduct ions of RDX, [RDX+NO_2_]– and [RDX+NO_3_]^−^, were observed as major ions using atmospheric-pressure DBD ion source (Na et al., 2007b; Garcia-Reyes et al., 2011; Habib et al., 2014; Bi et al., 2021).

Figures 13B,B_1_ show the mass spectra for 10 ng of PETN measured by HCD in negative ion mode at 5 and 28 Torr of ion source pressures, respectively. As shown in Figure 13B, the intensity of the fragment ion, [PETN-CH_2_ONO_2_]^−^ (*m/z* 240), for PETN is higher than that of the adduct ion with ${\text{NO}}_{3}^{-}$, [PETN+NO_3_]^−^ (*m/z* 378) at 5 Torr. The intensity of the adduct ion, [PETN+NO_3_]^−^, increases with ion source pressure and ultimately became as the major ion at 28 Torr. McLuckey et al. (1996) found only the fragment ion for PETN, [PETN-CH_2_ONO_2_]^−^ (*m/z* 240), in the negative ion mode using dc glow discharge through adjusting the ion source pressure at 0.8 Torr with air (McLuckey et al., 1996). However, by using the atmospheric pressure DBD ionization, PETN gave the adduct ion, [PETN+NO_3_]^−^ (*m/z* 378), as the major ion (Zhang et al., 2009; Habib et al., 2014), that was also found for the RDX (Figure 13A_1_).

Among the explosives, TNT has widely been studied through fabrication of various ion sources for mass spectrometry including MS^n^ system (Harper et al., 2008; Zhang et al., 2009; Nilles et al., 2010; Garcia-Reyes et al., 2011; Habib et al., 2013). Figures 13C,C_1_ show the negative mode mass spectra for 2 ng TNT measured by HCD ion source at 5 and 28 Torr, respectively. As seen from Figure 13C, the fragment ion, [TNT-H]^−^ (*m/z* 226), is the major ion at 5 Torr along with other fragment ions such as, [TNT-NO]^−^ (*m/z* 197), [TNT-2NO]^−^ (*m/z* 167, [TNT-3NO]^−^ (*m/z* 137) as the minor ions, however, TNT usually gives its molecular ion, [TNT]^−^, as the major ion even in the ambient conditions (Habib et al., 2013). Furthermore, TNT gave an adduct ion, [TNT-H+O]^−^ (*m/z* 242), as the minor ion at 5 Torr. The adduct ion, [TNT-H+O]^−^ (*m/z* 242), became as the major ion at 28 Torr (Figure 13C_1_). On the basis of the mass spectra of TNT at 5 and 28 Torr, it may conclude that the TNT molecules suffered from complicated ion-molecule reactions in the HCD ion source.

To investigate the formation of the fragment and adduct ions from TNT molecules using the HCD ion source, tandem mass spectrometry (MS^2^) was applied to monitor the collision induced dissociation (CID) products. Herein, the adduct ion, [TNT-H+O]^−^ (*m/z* 242), was taken as a precursor ion to monitor the MS^2^ CID product ions. The inset in Figure 13C_1_ shows the MS/MS product ion spectrum for the precursor ion, [TNT-H+O]^−^ (*m/z* 242). As seen from MS/MS spectrum (inset in Figure 13C_1_), the first product ion, *m/z* 225, is due to elimination of OH from the precursor ion, [TNT-H+O]^−^ (*m/z* 242), ion at *m/z* 210 is due to elimination of NO from [TNT]- (*m/z* 227), ion at *m/z* 167 is for elimination of NO from [TNT-NO]^−^ (*m/z* 197), ion at *m/z* 137 is for elimination of NO from [TNT-2NO]^−^ (*m/z* 167) and ion at *m/z* 107 is for elimination of NO from [TNT-3NO]^−^ (*m/z* 137).

Nitroglycerine (NG) an explosive compound was also analyzed by the HCD ion source in the negative mode of operation at 1, 5, and 28 Torr of ion source pressures (data not shown). Unlike RDX and PETN, NG did not show any ion signal at low ion source pressure such as 1 and 5 Torr even though the amount of NG was increased up to 10 ng, however, the adduct ion for NG with ${\text{NO}}_{3}^{-}$, [NG+NO_3_]^−^ (*m/z* 289), was observed at 28 Torr (Habib et al., 2015). In the negative ion mode, NG may provide ${\text{NO}}_{3}^{-}$ as a fragment ion (McLuckey et al., 1996; Hiraoka et al., 2010) that hampers the analysis of NG at low ion source pressure. This is because ${\text{NO}}_{3}^{-}$ is one of the reagent ions of the HCD ion source (see Figure 11B). Thermal instability of NG causes poor detection sensitivity as its adduct ion with the ${\text{NO}}_{3}^{-}$, [NG+NO_3_]^−^. Yinon et al. (1997) also found the formation of the adduct ion of NG with ${\text{NO}}_{3}^{-}$ reagent ion, [NG+NO_3_]^−^ (Yinon et al., 1997). To investigate the stability of NG, a preliminary experiment was done through depositing a considerable amount on a metal substrate and found it decomposed above ~100°C.

**Figure 14:**
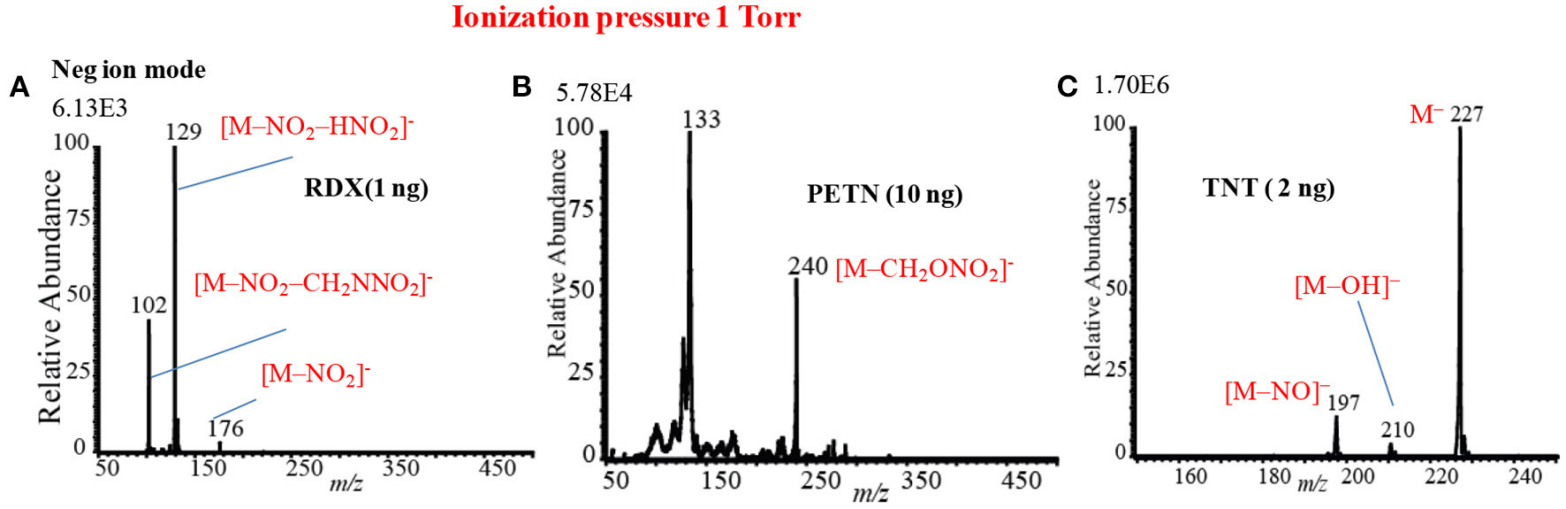
Mass spectra for **(A)** RDX (1 ng), **(B)** PETN (10 ng), and TNT (2 ng) at 1 Torr using the HCD ion source in the negative ion mode. Reproduced under permission from Wiley (Habib et al., 2015).

As seen from Figures 12, 13, the mass spectra for the explosive compounds remarkably depend on the ion source pressures. Thus, additional experiments for the explosives were performed at relatively lower ion source pressure (1 Torr) in order to investigate the ionization mechanism of the explosives with the ion source pressures. At 1 Torr, a dc plasma was generated between the transfer tube and S-lens by applying +0.85 kV and −0.85 kV in the positive and negative ion modes, respectively. In the both positive and negative ion modes, TATP and HMTD did not give any ion signals under 1 Torr ion source pressure, on the other hand, RDX, PETN, and TNT exhibited strong ion signals in the negative mode of operation (see Figures 14A–C). As seen from Figure 14A, RDX gave the fragment ion, [RDX-NO_2_-HNO_2_]^−^ (*m/z* 129) that found as the major ion along with the other fragment ions such as, [RDX-NO_2_-CH_2_NNO_2_]^−^ (*m/z* 102) and [RDX-NO_2_]^−^ (*m/z* 176) as the minor ions at 1 Torr. The results were in good agreement with the results obtained by McLuckey et al. (1996) where they used low pressure glow discharge ion source (0.8 Torr of air pressure) (McLuckey et al., 1996). Figure 14B shows the mass spectrum for 10 ng of PETN in the negative ion mode. Herein, PETN only gave the fragment ion, [PETN-CH_2_ONO_2_]^−^ (*m/z* 240) in the negative mode. McLuckey et al. (1996) also found the appearance of the fragment ion, [PETN-CH_2_ONO_2_]^−^, as the major ion at 0.8 Torr of air pressure (McLuckey et al., 1996). It is noted that RDX and/or PETN did not give an adduct ions such as [M+NO_2_]^−^ or [M+NO_3_]^−^ when the ion source pressure was kept at 1 Torr, however, these adduct ions were appeared in abundance at 5 and/or 28 Torr. Figure 14C shows the mass spectrum of TNT (2 ng) in the negative ion mode at 1 Torr. As seen from Figure 10C, TNT gave the molecular ion, [TNT]^−^ (*m/z* 227) as the major ion with very high ion intensity (1.70E6) under 1 Torr ion source pressure along with the [M-NO]^−^ (*m/z* 197) and [M-OH]^−^ (*m/z* 210) ions as the minor ions. The other fragment ions such as [M-H]^−^ (*m/z* 226), [M-2NO]^−^ (*m/z* 167) and also the adduct ion, [M-H+O]^−^ (*m/z* 242) were completely absent at 1 Torr (see Figure 14C). The major ions observed under different ion source pressures such as 1, 5 and 28 Torr are tabulated in Table 2. The limits of detection (LODs) with S/N ≥3 for the explosives of interest are also summarized in Table 2. It is noted that the explosive compounds did not give any dimer ions by using the fabricated HCD ion source.

On the basis of the analytical calibration curves, the values of LOD for HMTD, RDX, PETN, and TNT were 20, 40, 800, and 2 pg, respectively. These values are comparable to those obtained by DESI (Takáts et al., 2005; Cotte-Rodríguez et al., 2008), EESI (Chen et al., 2009), DBDI (Harper et al., 2008; Zhang et al., 2009; Garcia-Reyes et al., 2011), and ac-APCI (Habib et al., 2013). The LOD value for PETN was 800 pg measured by the HCD (Table 2) while Zhang et al. (2009) found only 500 fg using DBD ion source that is 16 times lower than that observed by the HCD ion source (Zhang et al., 2009). This is logical because the ions generated by the DESI, EESI and DBDI under ambient conditions and introduced directly into the mass spectrometer, thus, the compounds of interest and their ions do not suffer from thermal decomposition. On the other hand, in the HCD ion source, the formed ions might be suffering from the thermal decomposition because of the high temperature (150°C) of the ion source that was observed in this study (Table 2). The compact size of the HCD ion source also causes the neutralization of the formed ions by hitting walls of the ion source under reduced pressure. As seen from Figures 13, 14, the intensities (digits at left top in each spectrum) of the observed ions were increased with increased the ion source pressure except for TNT at 1 Torr where TNT was observed as its molecular ion, [TNT]^−^, with the strongest ion signal (ion intensity 1.70E6). Under low ion source pressure, the electron capturing process became more favorable for TNT molecules because of their positive electron affinity (Habib et al., 2014). As described above, lower ion source pressure is suitable to detect TNT with better sensitivity in the negative mode of operation, however, RDX, PETN, and NG in the negative ion mode and TATP and HMTD in the positive mode exhibited better sensitivity with low values of LOD under the higher ion source pressures. In the previous study, ion formation mechanism for the explosive compounds by using the HCD ion source has been significantly discussed (Habib et al., 2015).

Nitro- and nitro-groups containing compounds exhibit positive electron affinity, thus electron affinity for NO_3_ is 3.92 eV (Lias et al., 1988) while that for TNT is only 2.50 (Habib et al., 2015). Cooper et al. (2012) performed ab initio calculations in order to obtain the values of the electron affinities for RDX, HMX, TNT, and PETN and they found positive electron affinities for these explosive compounds. However, these explosive compounds did not give the molecular ion, M^−^, except TNT in the negative mode of operation (Habib et al., 2015). The results suggest that RDX, PETN and NG prefer adduct/cluster ion formation with the reagent ions i.e., ${\text{NO}}_{2}^{-}$/${\text{NO}}_{3}^{-}$ rather than electron capturing process in the HCD ion source. The results were in good agreement by the other groups where they used plasma-based ionization methods (Na et al., 2007b; Zhang et al., 2009; Garcia-Reyes et al., 2011; Habib et al., 2014). It has been reported that NO_x_ is one of the major products in plasma-excited air (Nagato et al., 2006; Sekimoto et al., 2012). The nitro-species (NO_x_) formed in the plasma-excited air capture electrons preferably because of their higher electron affinity than that of the relevant explosives, thereby resulting in decreasing the signal intensity of the [TNT]^−^ ion with pressure (Habib et al., 2015).

**Figure 15:**
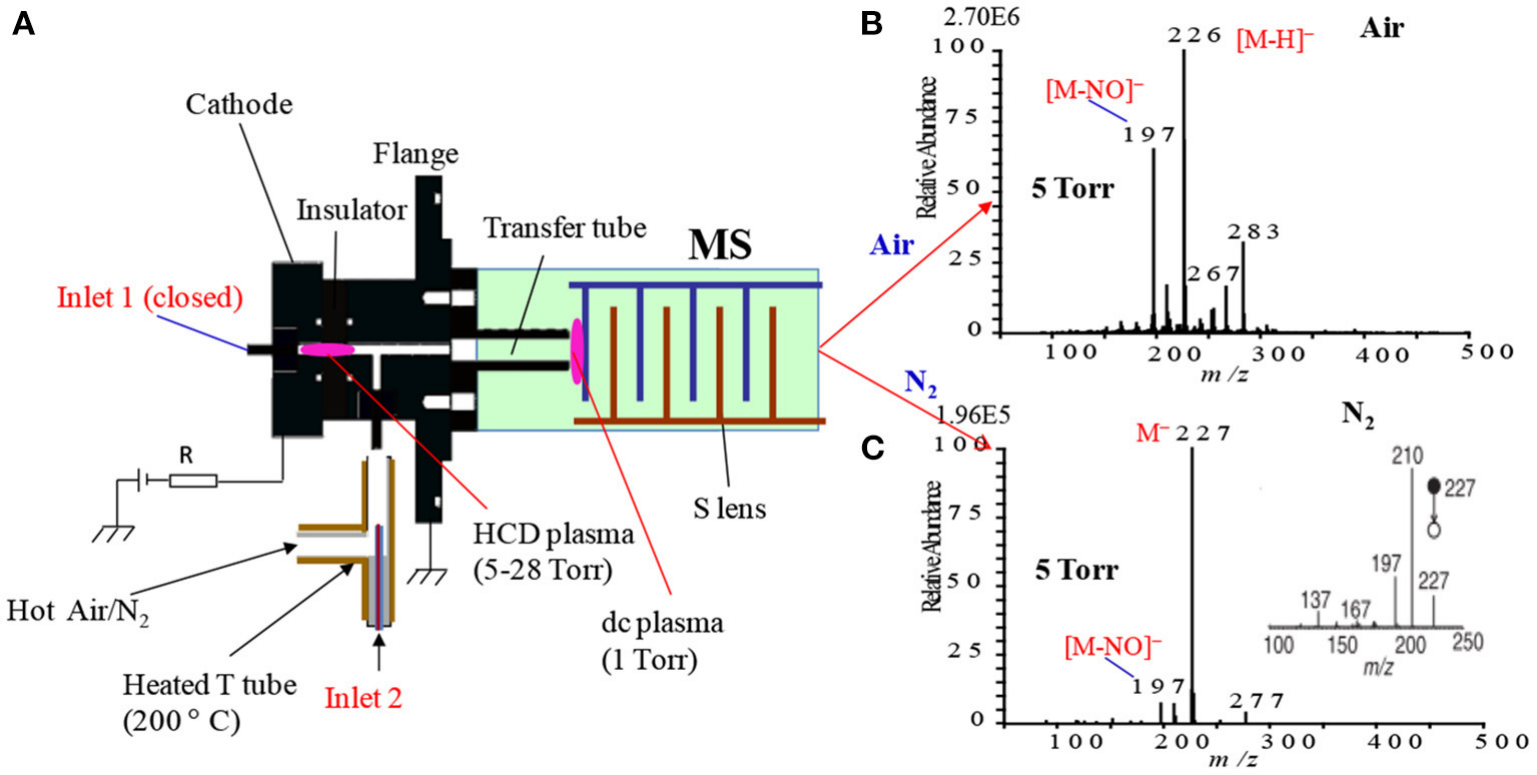
A diagram of the HCD ion source **(A)** (details are shown in Figure 10). Mass spectra of TNT using air **(B)** and N_2_ **(C)** as carrier gas in the negative ion mode under ion source pressure 5 Torr. The inset depicts the MS/MS mass spectrum for the TNT molecular ion, [TNT]^−^ (m/z 227), with 25% of CID. Reproduced under permission from Wiley (Habib et al., 2015).

As described above, ionization of TNT is strongly dependent on the ion source pressure (see Figures 13C,C_1_, 14C). To investigate the ion formation mechanism of the TNT molecules with ion source pressure, additional experiments were performed using air and nitrogen as carrier gas. Figure 15A shows a schematic of the fabricated HCD ion source in which there are two inlets: inlet-1 for introduction of the analytes with air into the ion chamber while inlet-2 was closed (see Figure 7). To confirm the ionization of TNT with different ion source pressures, a sample was introduced through inlet-2 while the inlet-1 was closed as shown in Figure 15A. Figures 15B,C show the negative HCD mass spectra of TNT for air and N_2_ as carrier gas, respectively. As seen from Figures 15B, there is no molecular ion of TNT, [TNT]^−^, as air was the carrier gas, but the fragment ions, [TNT-H]^−^ (*m/z* 226) and [TNT-NO]^−^ (*m/z* 197) are appeared with strong intensities where [TNT-H]^−^ is the major ion. In contrast, by using N_2_ as a carrier gas, the molecular ion for TNT, [TNT]^−^ (*m/z* 227) appeared as the major ion with a strong intensity where the fragment ion, [TNT-H]^−^ (*m/z* 226) has been completely disappeared (see Figure 15C). However, another fragment ion for TNT, [TNT-NO]^−^ (*m/z* 197) appeared as the minor ion with a much weaker intensity.

The explosive compound TNT has also been investigated by using different atmospheric pressure ion sources such as DBDI (Na et al., 2007b; Harper et al., 2008; Zhang et al., 2009; Chen et al., 2010; Usmanov et al., 2013), dc-corona APCI (Takada et al., 2002), electrospray-based techniques (Yinon et al., 1997; Takáts et al., 2005; Chen et al., 2009), and ac-corona APCI (Habib et al., 2013). By using the electrospray-based ionization methods, TNT usually gave its molecular ion, [TNT]^−^, as the major ion where the fragment ion, [TNT-H]^−^, rarely observed (Yinon et al., 1997; Takáts et al., 2005; Chen et al., 2009).

However, discharge-based ionization methods provided the strong ion signals of the fragment ion, [TNT-H]^−^, and molecular ion, [TNT]^−^ for TNT. Song and Cooks (2006) reported that TNT gave strong ion signals of the [TNT-H]^−^ and [TNT]^−^ using the desorption atmospheric pressure chemical ionization (DAPCI) (Song and Cooks, 2006). Dielectric barrier discharge ionization (DBDI) also produced the strong signals of the [TNT-H]^−^ and [TNT]^−^ (Na et al., 2007b; Zhang et al., 2009; Usmanov et al., 2013). Thus, it is concluded that ionization methods play a vital role in the relative ratio of [TNT]^−^ and [TNT-H]^−^. The presence of air in the plasma also causes the variation of the relative ratios of the [TNT]^−^ and [TNT- H]^−^ ions (Na et al., 2007b; Zhang et al., 2009; Usmanov et al., 2013; Habib et al., 2015). The direct analysis in real time (DART) exhibited a strong signal of the [TNT]^−^ ion because of use of helium plasma in DART (Cody et al., 2005; Nilles et al., 2010). The presence or absence of air in the plasma/glow causes the relative abundances of [TNT]^−^ and [TNT-H]^−^. It is, therefore, concluded that the analysis of explosives at trace-level is really a challenging task from the analytical point of view.

In summary, the developed HCD ion source exhibited better sensitivity for the explosives such as HMTD, TATP, RDX, PETN etc. both in the positive and negative ion modes under high ion source pressures, e.g., 5 and 28 Torr, however, ultra-trace level detection was achieved for TNT (LOD ~2 pg) at lower pressure (1 Torr) in the HCD plasma in the negative ion mode. At 1 Torr, no cluster ions were observed from the RDX and PETN through ion-molecule reactions while these two explosive compounds gave fragment ions with better sensitivity, particularly RDX at lower ion source pressure (1 Torr). It is noted that density of electrons goes to the maximum levels under low ion source pressure (e.g., 1 Torr) in the HCD plasma, thus compounds having positive electron affinity take part in electron attachment reactions. Accordingly, TNT exhibited its molecular ion, [TNT]^−^ (*m/z* 227), as the major ion through electron attachment reactions while RDX and PETN took part in electron capture dissociation in the negative ion mode at 1 Torr. The estimated value of electron affinity for TNT was found to be 0.6–0.7 eV (Batley and Lyons, 1962; Foster, 1969). While the electron capture dissociation reactions of RDX and PETN gave their fragment ions as the major ion signals under low ion source pressure (1 Torr). RDX and PETN gave cluster ions with the reagent ions i.e., ${\text{NO}}_{2}^{-}$, ${\text{NO}}_{3}^{-}$ at higher ion source pressure such as 5 and 28 Torr while AN showed cluster ion of HNO_3_ with ${\text{NO}}_{3}^{-}$, [HNO_3_+NO_3_]^−^ (*m/z* 125). This is because AN decomposes to HNO_3_ in the plasma-excited air and the formed HNO_3_ forms cluster ion with the predominantly present ${\text{NO}}_{3}^{-}$ ion in the HCD ion source. Among the explosive compounds, TNT showed significant pressure dependence mass spectra. At 1 Torr, the major ion signal for TNT was its molecular ion, [TNT]^−^ (*m/z* 227), however, fragment ions became major ions such as [TNT-H]^−^, [TNT-*n*NO]^−^ (*n* = 1–3), and [TNT-H+O]^−^ with increasing ion source pressure from 1 to 28 Torr. It has been proposed that the predominant presence of ${\text{NO}}_{2}^{-}$, ${\text{NO}}_{3}^{-}$ as well as O_3_ in the HCD ion source causes the formation of these fragment ions. The limits of detection for the explosives examined were a bit higher than those reported by DESI, DBDI and/or DAPCI, however, the present HCD ion source showed as robust, compact and easy to operation. Moreover, the use of air as a carrier gas in the HCD ion source is its merit to deploy at public places in order to detect explosives along drugs of abuse.

## Alternating Current Corona Discharge/Atmospheric Pressure Chemical Ionization (ac-APCI)

In the atmospheric pressure chemical ionization (APCI), direct current (dc) has long been used since its pioneering work by Horning and co-workers in the 1970s (Horning et al., 1973). In 1975, Carroll and co-workers developed a corona discharge electrode, which had a larger dynamic response range (Carroll et al., 1975). The APCI has already been coupled to mass spectrometry and has widely been used to the commercial GC/MS (Horning et al., 1973) as well as to LC/MS (Horning et al., 1974; Byrdwell, 2001).

**Figure 16:**
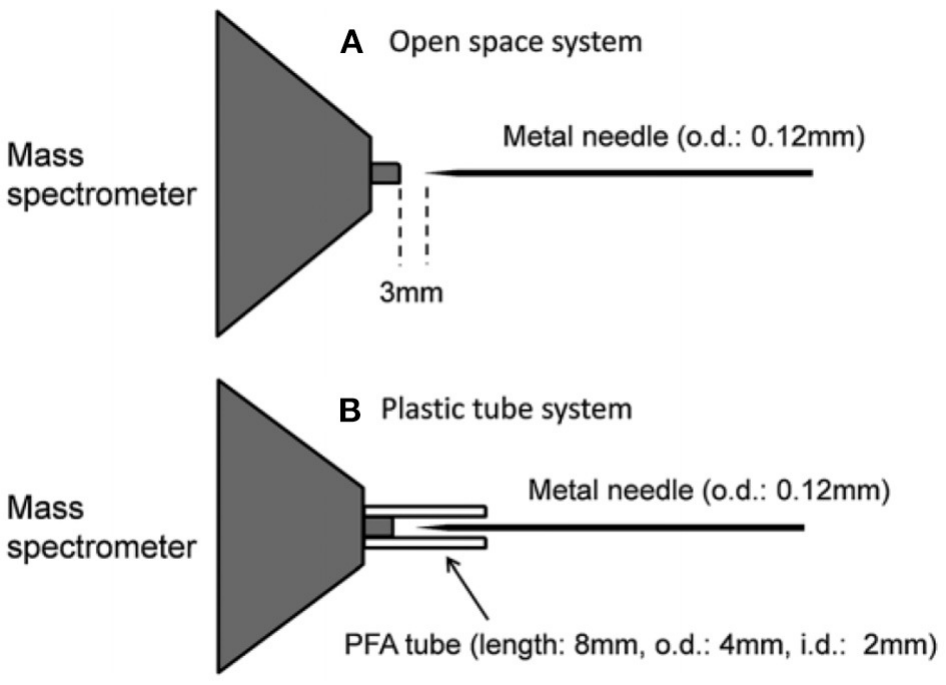
Experimental set ups for ac- and dc corona-based APCI. A stainless steel made acupuncture needle, having 0.12 mm of o.d. and 700 nm of tip diameter, was used as an electrode. The needle was positioned at 3 mm far from the inlet of the mass spectrometer. **(A)** for open space system and **(B)** for plastic (PFA) tube with 8 mm long and its i.d.: 2 mm and o.d.: 4 mm. Reproduced under permission from Wiley (Habib et al., 2013).

An ac-corona-based ambient atmospheric pressure chemical ionization (ac-APCI) was fabricated using an acupuncture needle (stainless steel made) with o.d. (outer diameter); 0.12 mm and tip diameter: 700 nm (Seirin, Shizuoka, Japan). For comparison, the same acupuncture needle was used to generate dc-corona as well. Figure 16 shows the experimental setup for ac- and dc corona-based APCI. As seen from Figure 16, the distance between the needle and inlet of the MS was only 3 mm. Figures 16A,B show open space and closed system plastic tube (perfluoroalkoxy, PFA), respectively.

In the fabrication of ac corona-based APCI, a function generator was used to generate 15 kHz radio frequency (RF) in order to apply ac voltage to the needle. Exactly 2.6 kV_PTP_ (peak-to-peak) ac voltage was applied to the needle without plastic tube while 2.7 kV_PTP_ (peak-to-peak) was for the needle with the plastic tube. In the dc corona-based APCI, a range of voltage from +2.5 to −1.5 kV was applied to the needle without plastic in the positive and negative ion modes while that ranging from +3.4 to −2.3 kV was applied to the needle with plastic, respectively through 4 μA discharge current kept constant. Due to the charging effect inside the plastic tube, the dc discharge voltage was fluctuated and found relatively higher values than that for ac corona with plastic. The voltages for the ac and dc corona were adjusted just above the threshold levels of gas discharge in order to obtain stable glow/plasma. The high voltage causes arc or spark discharge that leads to generation of extremely high discharge currents (≥200 μA) (Akishev et al., 2005). The generation of arc between the needle and the counter electrode (metallic flange of MS) may cause damage to the MS through creating a short circuit.

Triacetone triperoxide (TATP) and TNT were taken as model explosive compounds to evaluate the performance of the developed ac-APCI as an ambient ion source for trace-level detection of explosives. Herein, the developed ac-APCI was compared with a dc-APCI using the TATP and TNT in positive and negative ion mode, respectively in order to investigate its potentiality as an alternative to commercial dc-APCI (Habib et al., 2013). The electric field created by the plasma at the tip of the needle is radially diffused along the electric field for the open system as shown in Figure 16A, however, in the closed system (with plastic tube), the plasma was confined in the plastic tube (Figure 16B). In fact, the charging effect created inside the plastic tube causes it to confine the plasma inside the tube. The different characters of the dc plasmas without and with plastic tube leaded characteristics spectral pattern from the relevant explosive compounds.

### Positive Ion Mode AC- and DC Corona-Based APCI

**Figure 17:**
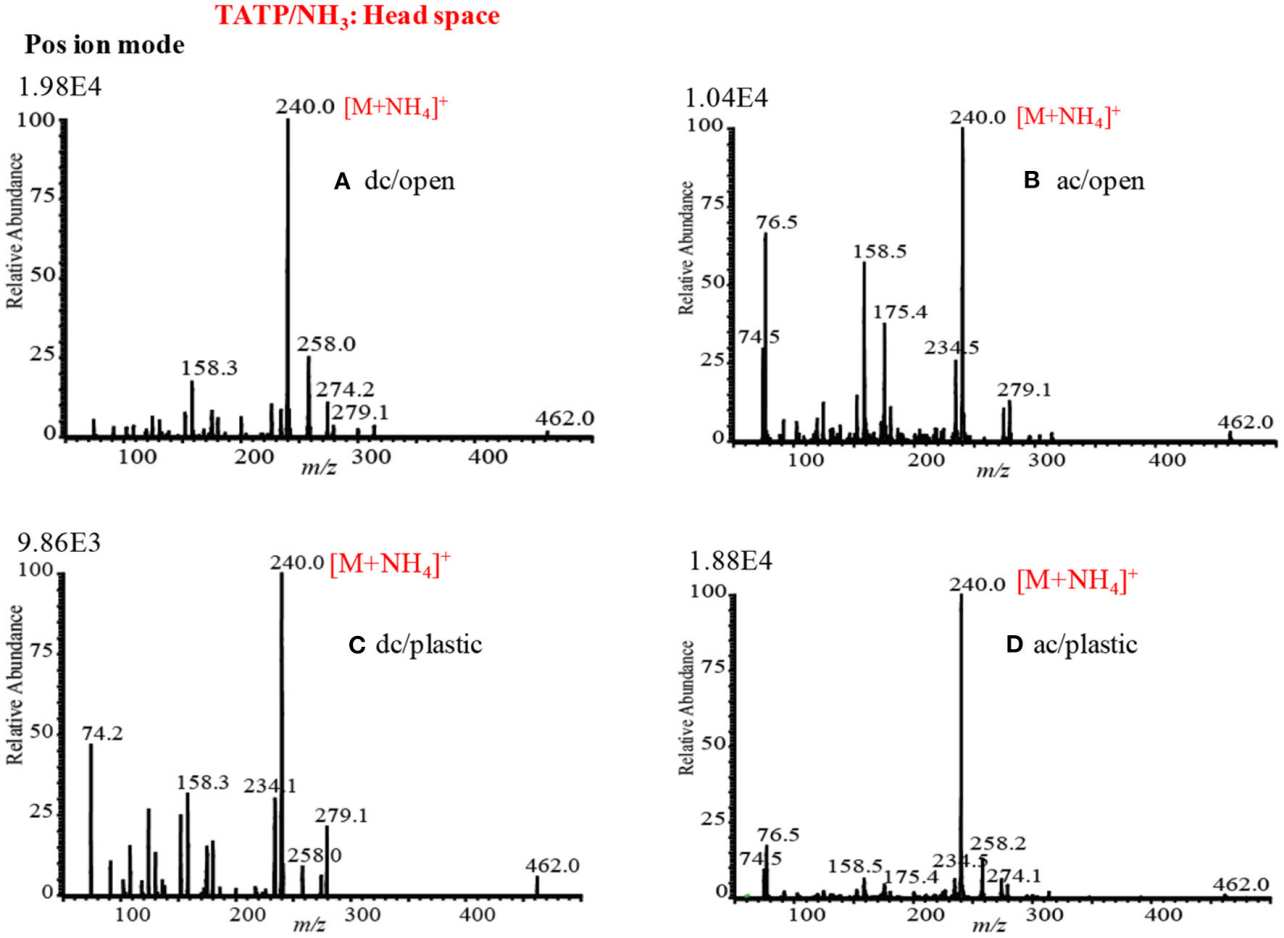
Mass spectra of TATP (head-space gas) in the positive ion mode **(A)** dc-APCI without plastic tube (applied voltage: +2.5 kV dc), **(B)** ac-APCI without plastic tube (applied voltage: 2.6 kVptp ac), **(C)** dc-APCI with plastic tube (applied voltage: +3.4 kV dc), and **(D)** ac-APCI with plastic tube (applied voltage: 2.7 kVptp ac). Reproduced under permission from Wiley (Habib et al., 2013).

Figure 17 shows the mass spectra of TATP in positive ion mode by using ac/dc open and plastic tube systems. As seen from Figure 17, TATP showed an adduct ion with ammonium, [TATP+NH_4_]^+^, as the base peak for all the systems along with many background signals originated from the laboratory air. This result suggests that ac corona (Figures 17B,D) can be used in the development of APCI ion source for mass spectrometry because of similar performance to the dc corona discharge (Figures 17A,C).

Sigman et al. (2006) reported that TATP also forms adduct ion with ammonium, [TATP+NH_4_]^+^ by using electron and chemical ionization coupled to GC-MS and/or GC-MS/MS systems, however, they did not observe both the fragment ions (having *m/z* values higher than 60) and protonated molecular ion, [TATP+H]^+^ (*m/z* 223), through applying subsequent collision-induced dissociation (CID) (Sigman et al., 2006). The binding energy for the [TATP-NH_4_] was calculated using DFT (basis set: B88LYP/DVZP level) calculation and found to be 25 kcal/mol.

As seen from Figures 17A–D, TATP gave strong ion signal as its ammoniated adduct ion, [TATP+NH_4_]^+^ (*m/z* 240), in all types of ac/dc and open/plastic system in the positive ion mode. The appearance of the adduct ion, [TATP+NH_4_]^+^ (*m/z* 240), as the major ion would be used as a better diagnostic ion at trace-level detection of TATP at public places in order to provide public security and safety. In the open system, the intensity of the adduct ion was about two times higher in dc/open than that in ac/open. Contrast to the closed system (with plastic tube), the intensity of the adduct ion was about two times higher for ac/closed compared to that for dc/closed system. The creation of static charge inside the plastic tube traps the positive adduct ion, [TATP+NH_4_]^+^, that leads to the lower value of the ion intensity. It is expected that the value of the static potential (wall potential) would be higher than that of plasma, thus the adduct ion, [TATP+NH_4_]^+^, suffered from fragmentation. This could be the reason for observing the low value of the intensity of the adduct ion in the dc/plastic system (Habib et al., 2013).

### Negative Ion Mode AC- and DC Corona-Based APCI

**Figure 18:**
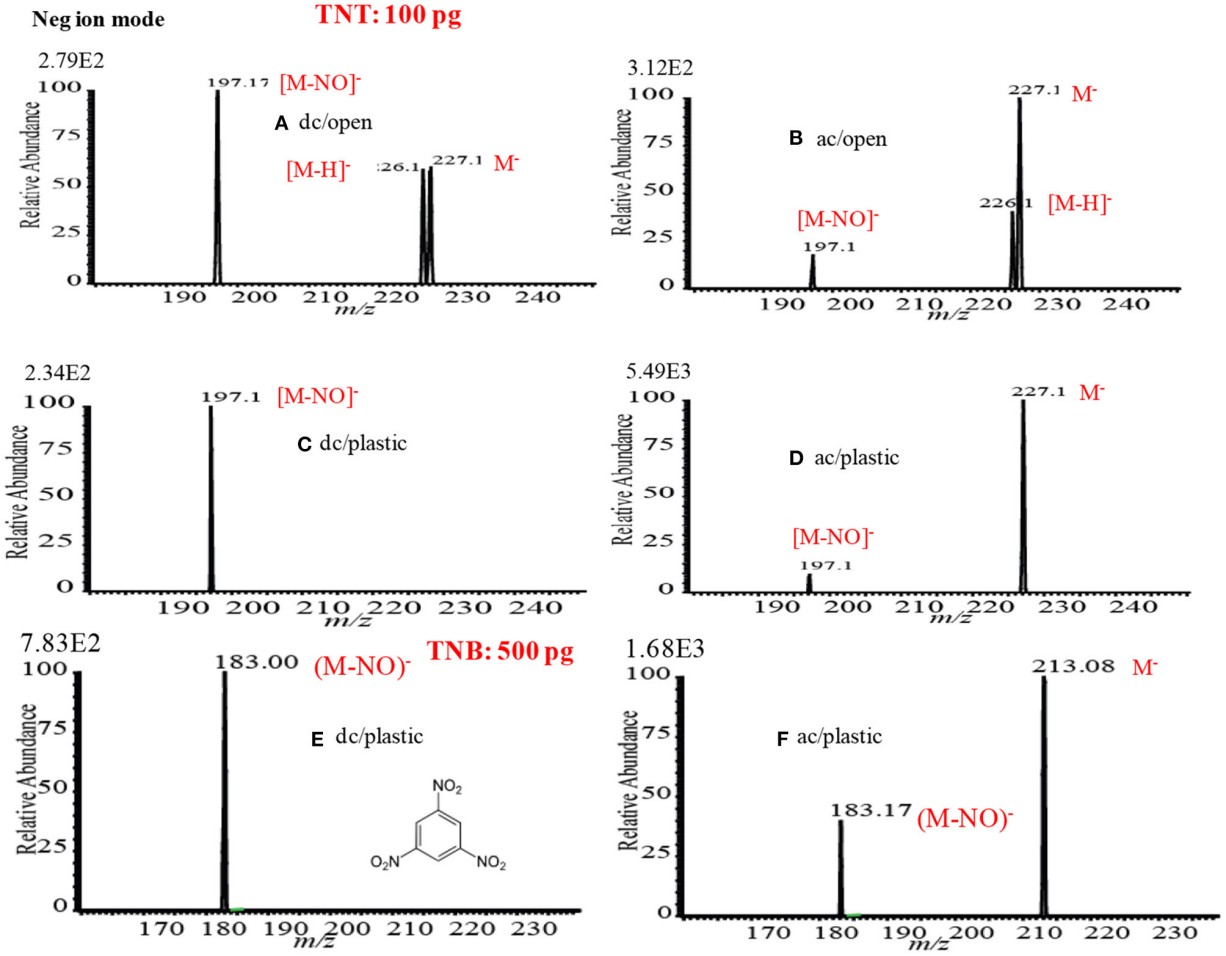
Mass spectra of TNT in the negative ion mode **(A)** dc-APCI without plastic tube (dc applied voltage: −1.5 kV), **(B)** ac-APCI without plastic tube (ac applied voltage: 2.6 kVptp), **(C)** dc-APCI with plastic tube (applied voltage: −2.3 kV dc), **(D)** ac-APCI with plastic tube (applied voltage: 2.7 kVptp ac), **(E)** TNB, dc-APCI with plastic tube (applied voltage: −2.3 kV dc), and **(F)** TNB, ac-APCI with plastic tube (applied voltage: 2.7 kVptp ac). Reproduced under permission from Wiley (Habib et al., 2013).

As mentioned above, TNT was taken as a model explosive compound to investigate the performance of the fabricated ac- and dc corona-based APCI. Figures 18A,B show the mass spectra of TNT by using dc/open-APCI and ac/open-APCI, respectively. As seen from Figure 18A, TNT gave the fragment ion, [TNT-NO]^−^ (*m/z* 197), as the major ion while the molecular ion, [TNT]^−^ (*m/z* 227), and/or fragment ion, [TNT-H]^−^ (*m/z* 226), appeared as the minor ions. Contrast to the ac/open APCI, TNT rather gave its molecular ion, [TNT]^−^, as the major ion where the fragment ion, [TNT-NO]^−^, along with the adduct ion, [TNT-H]^−^, appeared as the minor ions (Figure 18B). These results suggest that the ion-molecule reactions for TNT molecules took place rather softer in the ac/open APCI.

As seen from Figure 18C, TNT gave only the fragment ion, [M-NO_2_]^−^ (*m/z* 197), in the dc/plastic APCI system. However, TNT exhibited its molecular ion, [TNT]^−^, as the major ion while the only fragment ion, [TNT-NO]^−^, appeared as the minor ion with a much weaker intensity in the ac/plastic APCI system. But it was expected to observe the fragment ion, [TNT-H]^−^, under the ambient condition for the ac/plastic system. This result suggests that TNT took place in an electron capturing reaction in the ac-corona that leads to the formation of its molecular ion, [TNT]^−^. It is, therefore, concluded that the ac/plastic-based APCI showed as a much softer ion source than the dc/plastic-APCI. In order to confirm this phenomenon, 1,3,5- trinitrobenzene (TNB), which is structurally similar to TNT, was investigated using the dc/plastic and ac/plastic systems and its mass spectra are shown in Figures 18E,F, respectively. As seen from Figure 15E, TNB gave only the fragment ion, [M-NO]^−^ (*m/z* 183) that is just mimic of TNT in the dc/plastic system while TNB showed its molecular ion, [TNB]^−^ (*m/z* 213), as the major ion where the fragment ion, [TNB-NO]^−^ (*m/z* 183) as the minor ion in the ac/plastic system.

The developed dc/plastic-APCI and ac/plastic-APCI ion sources were examined in order to evaluate their analytical validation in quantification of explosives at trace level. Herein, TNT was taken as a model explosive compound. In the dc/plastic system, signal intensities for the fragment ion, [M-NO]^−^ (*m/z* 197) were used to construct the analytical calibration curve while that for the molecular ion, [TNT]^−^ (*m/z* 227), were used for the calibration curve in the ac/plastic system. A good dynamic range up to 2 ng was observed for both the cases with a correlation coefficient *R*^2^ of ~0.99 (Habib et al., 2013). The values of limits of detection (LODs) for TNT (S/N: 3) were 50 and 30 pg in the dc/plastic and ac/plastic systems, respectively. The low values of standard deviations with five replications demonstrate the good reproducibility in the dc/plastic and ac/plastic systems.

As seen from Figures 18C,E, the ions were underwent severe fragmentation in the dc/plastic compared to that in the ac/plastic for TNT and TNB, respectively while dc/open system exhibited moderate and ac/open showed less fragmentation in the negative mode of ionization. The PFA plastic surface acts as perfectly an insulator, thus the applied high voltage creates static charging in the inner wall of the plastic tube (Akishev et al., 2005). It is expected that the plasma developed inside the plastic tube distributed uniformly and becomes convex at the tip of the needle that causes the plasma to be diffusive toward the radial direction in the dc/open system. Under this circumstance, the formed primary ions in the plasma have less chance to undergo secondary decomposition (Habib et al., 2013). On the contrary, a concave shape of the equipotential surface is formed at the tip of the needle because of the inner wall static charging. The formation of the concave shape allows the confinement of the plasma within a narrow space, thus the ions generated must undergo further severe secondary fragmentation. The ac corona, however, should be different from the dc corona from their origin point of views. The ac corona plasma generated in the plastic (PFA) tube must have some characteristic features compared to that generated in the open system. The inner insulating wall has a strong effect on the local field distortion caused by a surface charge accumulation on the dielectric (Akishev et al., 2005; Habib et al., 2013). The charge accumulation developed on the dielectric surface controls the discharge current, thus the initially formed plasma ceases before the transition from glow discharge to arc-like hot plasma. The wall charging causes the occurrence of a non-equilibrium cold plasma that is responsible for the better performance in the ac/plastic system as shown in Figures 18D,F.

**Figure 19:**
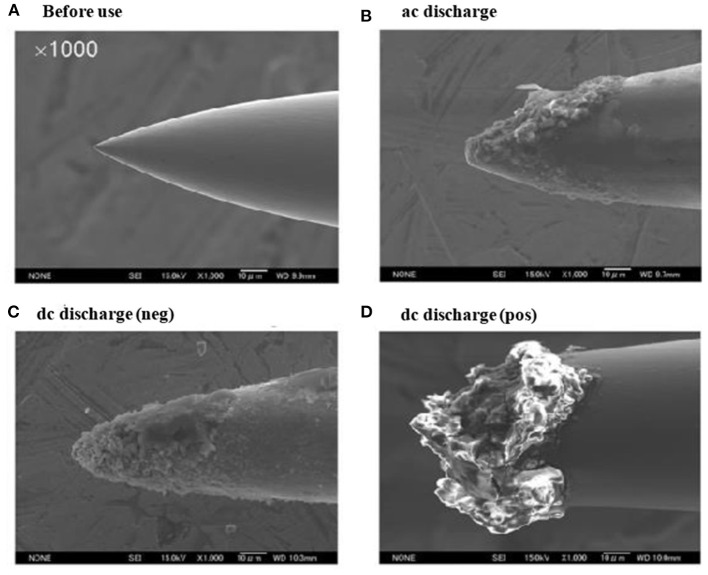
Scanning electron microscope (SEM) images of acupuncture needles made of stainless steel: **(A)** before use, **(B)** ac corona discharge in open space for 20 h (applied voltage: 2.6 kVptp ac), **(C)** –dc corona discharge in open space for 20 h (applied voltage: 1.5 kV dc), and **(D)** +dc corona discharge in open space for 20 h (applied voltage: +2.5 kV dc at the start and +2.8 kV dc after 20 h). Reproduced under permission from Wiley (Habib et al., 2013).

As described above, dc corona in positive as well as in negative ion modes showed some different features. In our previous study, we found that the applied voltage for the negative mode (-1.5 kV) was lower than that for the positive mode (+2.5 kV) in the open system in order to keep a constant current of 4 μA (Habib et al., 2013). It is noted that a 4 μA current was kept constant throughout the dc mode experiments. The low value of the applied voltage in the negative mode of operation than that of the positive mode is needed to maintain stable plasma in the negative mode. It is highly expected that the value of the applied voltage in the negative mode of dc corona would be low because of gaining additional electrons by the photoelectron emission and the value was found to be −1.5 kV (Akishev et al., 2003; Habib et al., 2013). Contrary to the dc in positive mode, the applied voltage to the needle tip varied from the initial value of +2.5 kV to the higher values up to +2.8 kV to maintain the constant discharge current. To compare the ac corona-APCI as a soft ionization with the dc corona-APCI ion source, the used acupuncture needles in the open system were examined for erosion by using scanning electron microscope (SEM) and energy dispersive X-ray spectroscopy (EDS) spectra. Figures 19A–D show the SEM images of the used acupuncture needles for ac and dc discharges in the open system. The SEM image for the pristine acupuncture needle is shown in Figure 19A while Figures 19B–D stand for the SEM images of the used in the ac corona, dc negative and dc positive corona after 20 h of continuous discharge operation, respectively. As seen from Figures 19B,C, the tip of the needles was corroded slightly, but the original shape of the needles was reasonably maintained while the tip of the needle was completely eroded and some rugged product was formed at the tip in the positive dc mode of operation (Figure 19D). In the dc positive mode, the voltage was applied to a point electrode but extended to a much longer distance from the point toward the gap than for the negative corona (Lowke and Morrow, 1994). This is because the discharge is streamer discharge and it has two modes such as diffusive and filamentary streamer mode. In the filamentary streamer mode, the surface of the electrode gains the joule energy, thus the tip of the needle becomes heated (Akishev et al., 2003). The heating effect causes severe erosion of the needle rather in the positive dc corona (Bárdos and Baránková, 2009). Therefore, it is concluded that the streamer discharge leads to the melting of the tip of the needle at the filamentary discharge spot in the positive dc corona. However, a thicker needle has widely been used to commercial dc corona APCI.

**Figure 20:**
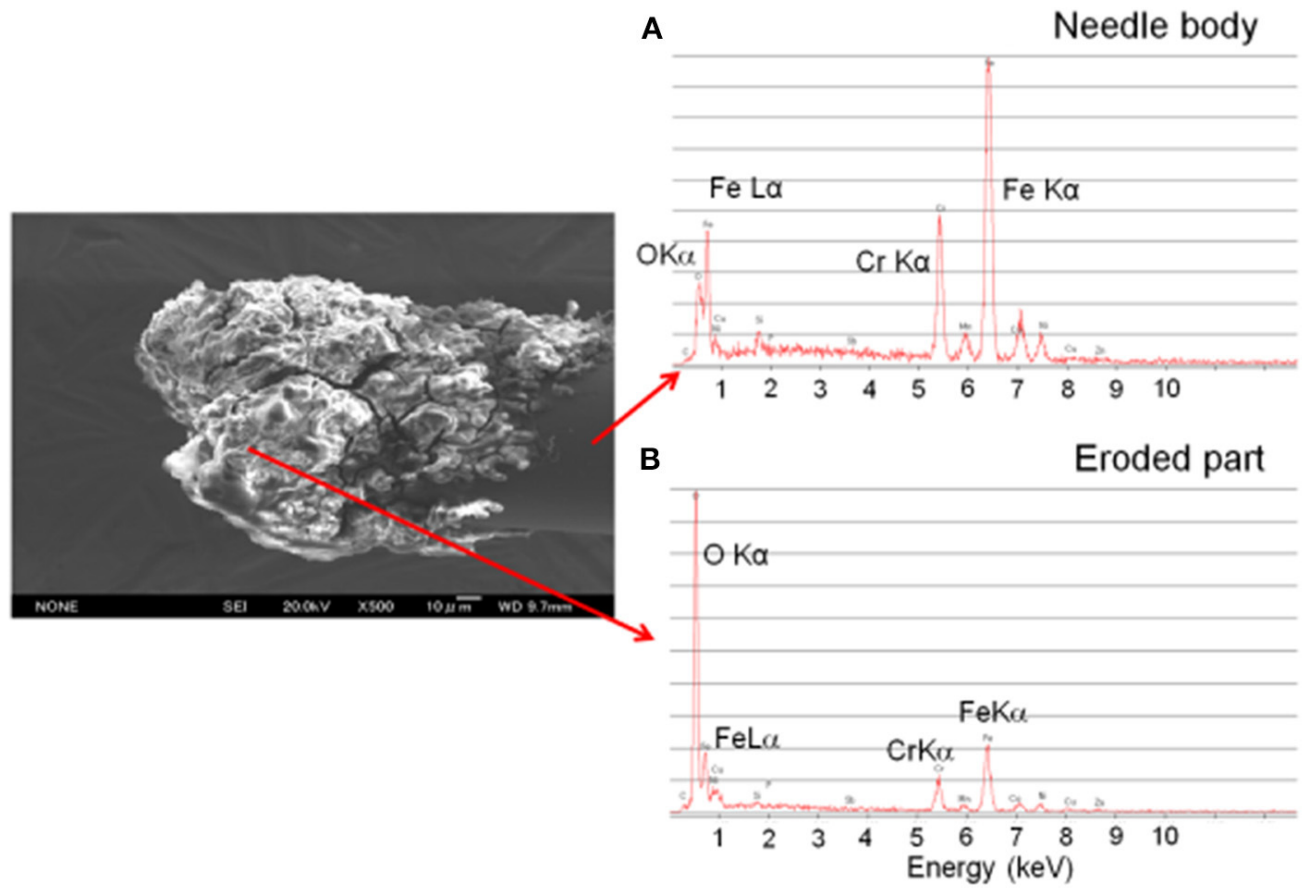
Energy dispersive X-ray spectroscopy (EDX) spectra for the stainless steel acupuncture needle: **(A)** needle body and **(B)** eroded part. Reproduced under permission from Wiley (Habib et al., 2013).

Figures 20A,B show the EDX spectra for non-eroded and eroded part of the used acupuncture needle, respectively. As seen from Figure 20A, the signal intensity of the iron Kα from the body part was much stronger than that of the oxygen Kα. However, the Kα for the oxygen was found as the highest intensity from the eroded part as shown in Figure 20B. This result is confirming the formation of oxides at the tip of the acupuncture needle for the dc positive mode of operation. The appearance of the second highest intensity for chromium Kα is reasonable. This is because stainless steel is an alloy of iron with mostly chromium (~18%). When chrome alloy exposes to air, its surface metallic chromium converts to Cr_2_O_3_ and forms a very thin layer. The formation of a thin layer of the Cr_2_O_3_ prevents the oxidation of iron in steel, however, the tip of the acupuncture needle becomes melted in the positive dc corona discharge mode. This is because the high energetic ejected electrons return back to the needle in the positive dc polarity and heated up the tip of the needle and then melted. The melted part of the needle is readily exposed to air, thereby resulting in formation of oxides of Fe and Cr. Therefore, it is reasonable to observe the very high intensity of oxygen Kα from the eroded part of the needle.

An atmospheric pressure chemical ionization (APCI) ion source has been fabricated with the means of point-to-plane ac corona discharge in order to detect explosives. Here, TATP and TNT were taken as model compounds for positive and negative modes of operation, respectively. The developed ac corona APCI gave ion signals that were as strong as in the usual dc corona-based APCI. A perfluorinated plastic tube (PFA) was used as an insulation tube to investigate the ionization mechanism for the explosive compounds. The ac and dc type corona discharges were examined with and without the plastic insulation tube in order to investigate the fragmentation mechanisms of the relevant explosives. Static charging was developed in the insulator wall and that causes fragmentation of the primary ions in the negative dc corona, however, this effect was minor in the ac corona discharge. The ac/plastic system was found to be the best for the APCI ion source. The needle for the ac corona was less eroded than that for positive mode dc corona and was found to be suitable for long-term operation.

## Concluding Remarks

In this review article, fabrication of new ion sources such as homemade helium-dielectric barrier discharge ionization (DBDI), hollow cathode discharge ionization (HCDI), ac-atmospheric pressure chemical ionization (APCI) for mass spectrometry has been taken into account in order to analyze drugs of abuse and explosives at trace to ultra-trace levels. Ultrasonic cutter blade-based desorption method for desorption of solid non-volatile drugs of abuse and explosives has also been discussed. The developed ultrasonic cutter blade-based desorption method showed as a non-thermal method for desorption of non-volatile compounds such as drugs of abuse, explosives etc. The desorption method is also exhibited as a soft desorption process for non-volatile compounds. This is because of the generation of mechanical frictional energy from the cutter blade that is much coherent compared to thermal energy. The developed desorption method is reasonably sensitive and may be applied for quick analysis of contaminants, such as narcotics, explosives and/ or any other non-volatile compounds, deposited on substrates' surfaces. The fabricated homemade He-DBDI ion source was found to ionize a wide range of drugs of abuse and explosives without and/or with minimal fragmentation. The pressure dependent HCD ion source exhibited as a suitable ion source for MS to detect the explosive compounds at trace to ultra-trace levels. The beauty of the fabricated HCD ion source is its carrier gas is air, thus this ion source may be commercialized through coupling with an MS in order to detect explosives at public places. The headspace method has been proven its versatile use in detection of amphetaminic drug compounds even from body fluids at ultra-trace levels. The ac corona-based APCI exhibited as a promising new ion source for MS in detection of explosives.

Despite profuse prospects in the field of MS-based analytical science, there are still some challenges in exploring the remarkable effectiveness of the developed new ion sources and new desorption methods for non-volatile compounds in future:

1. A single ion source should not be suitable for all types of the drugs of abuse and explosives.
2. Investigation of the coupling of the ultrasonic cutter blade-based desorption process with the homemade DBDI-MS system.
3. Fabrication of air-based DBD ion source for ultra-trace levels detection of the drugs of abuse and explosives.
4. Exploration of the developed ion sources for other compounds should be investigated
5. Development of hybrid ion source for MS in order to analyze a wide range of compounds.
6. The developed ultrasonic cutter blade-based desorption method needs further investigation in order to make it as a versatile desorption method for non-volatile compounds.

## Author Contributions

AH: conceptualization, writing original draft-review, and final review & editing. LB and HH: conceptualization and funding acquisition. LW: conceptualization, supervision, final review & editing, and funding acquisition. All the authors have read and agreed to the published version of the manuscript.

## Conflict of Interest

HH was employed by company China Innovation Instrument Co., Ltd, Ningbo 315000, Zhejiang, China. The remaining authors declare that the research was conducted in the absence of any commercial or financial relationships that could be construed as a potential conflict of interest.

## Abbreviations

MS: mass spectrometry
MS/MS: mass spectrometry/mass spectrometry
IMS: ion mobility spectrometry
GC: gas chromatography
LC: liquid chromatography
HPLC: high performance liquid chromatography
GC-MS: gas chromatography-mass spectrometry
LC-MS: liquid chromatography-mass spectrometry
HPLC-MS: high performance liquid chromatography-mass spectrometry
MSE: microfluidic surface extractor
DBDI: dielectric barrier discharge ionization
HCDI: hollow cathode discharge ionization
ac/dc-APCI-ac/dc: atmospheric pressure chemical ionization
ESI: electrospray ionization
Nano-ESI: nano-electrospray ionization
PESI: probe electrospray ionization
MALDI: matrix assisted laser desorption/ionization
DESI: desorption electrospray ionization
DART: direct analysis in real time
EESI: extractive electrospray ionization
EI: electron ionization
CI: chemical ionization
FAB: fast atom bombardment
PI: Panning ionization
TB: tribodesorption
AM: amphetamine
MA: methamphetamine
MDA: 3,4-methylenedioxyamphetamine
MDMA: 3,4-methylenedioxymethamphetamine
TATP: triacetone triperoxide
HMTD: hexamethylene triperoxide diamine
TNT: trinitrotoluene
TNB: 1,3,5-trinitrobenzene
AN: ammonium nitrate
RDX: hexahydro-1,3,5-trinitro-1,3,5-triazine
HMX: 1,3,5,7-tetranitro-1,3,5,7-tetrazoctane
NG: nitroglycerine
PETN: pentaerythritol tetranitrate
DFT: density functional theory
JAXA: Japan Aerospace Exploration Agency
ISRO: Indian Space Research Organization
NASA: National Aeronautics and Space Administration.
